# High Temperature Electron Diffraction on Organic Crystals: *In Situ* Crystal Structure Determination of Pigment Orange 34

**DOI:** 10.1021/jacs.3c14800

**Published:** 2024-03-27

**Authors:** Yaşar Krysiak, Sergi Plana-Ruiz, Lothar Fink, Edith Alig, Ulrich Bahnmüller, Ute Kolb, Martin U. Schmidt

**Affiliations:** † Institute of Inorganic Chemistry, Leibniz University Hannover, Callinstraße 9, 30167 Hannover, Germany; ‡ Department of Materials and Geoscience, Technische Universität Darmstadt, Petersenstrasse 23, 64287 Darmstadt, Germany; § LENS, MIND/IN2UB, Departament d’Enginyeria Electrònica i Biomèdica, Universitat de Barcelona, Martí i Franquès 1, 08028 Barcelona, Catalonia, Spain; ∥ Institute of Inorganic and Analytical Chemistry, Goethe University Frankfurt am Main, Max-von-Laue-Str. 7, 60438 Frankfurt am Main, Germany; ⊥ Institute of Inorganic Chemistry and Analytical Chemistry, Johannes Gutenberg University, Duesbergweg 10-14, 55128 Mainz, Germany

## Abstract

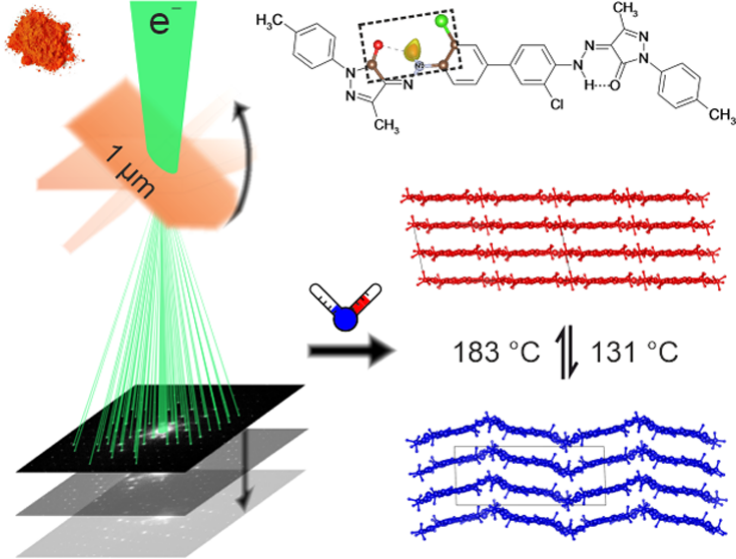

Small molecule structures and their applications rely on good knowledge of their atomic arrangements. However, the crystal structures of these compounds and materials, which are often composed of fine crystalline domains, cannot be determined with single-crystal X-ray diffraction. Three-dimensional electron diffraction (3D ED) is already becoming a reliable method for the structure analysis of submicrometer-sized organic materials. The reduction of electron beam damage is essential for successful structure determination and often prevents the analysis of organic materials at room temperature, not to mention high temperature studies. In this work, we apply advanced 3D ED methods at different temperatures enabling the accurate structure determination of two phases of Pigment Orange 34 (C_34_H_28_N_8_O_2_Cl_2_), a biphenyl pyrazolone pigment that has been industrially produced for more than 80 years and used for plastics application. The crystal structure of the high-temperature phase, which can be formed during plastic coloration, was determined at 220 °C. For the first time, we were able to observe a reversible phase transition in an industrial organic pigment in the solid state, even with atomic resolution, despite crystallites being submicrometer in size. By localizing hydrogen atoms, we were even able to detect the tautomeric state of the molecules at different temperatures. This demonstrates that precise, fast, and low-dose 3D ED measurements enable high-temperature studies the door for general *in situ* studies of nanocrystalline materials at the atomic level.

## Introduction

Structural changes in solids are frequently initiated by an external stimulus, such as temperature changes, pressure, irradiation, magnetic field, or chemical reactions. Structural changes in submicrometer sized materials could be investigated by, e.g., X-ray powder diffraction (XRPD) or by electron diffraction (ED). The information content of an ED pattern is much higher than of an XRPD pattern, because ED yields a full three-dimensional single-crystal diffraction pattern, whereas in XRPD the three-dimensional reciprocal space is projected onto one-dimensional diffraction data (usu. 2θ-axis) with overlapping reflections.

Three-dimensional electron diffraction (3D ED) is becoming increasingly important for the structure analysis of submicron-sized organic crystals, including pharmaceuticals,^1,2^ peptides and proteins,^3−7^ and organic pigments.^8−11^ In recent years, 3D ED had a real breakthrough toward a routine determination of crystal structures, even of beam sensitive materials. This breakthrough was mainly caused by two developments: (1) improved measurement strategies with different specific methods in each case^12−14^ that provide excellent and complete data, much better than the zonal electron diffraction patterns used hitherto (see Supporting Information S1);^15^ (2) the development of detectors with higher sensitivity and faster read-out times,^3^ which allow fast and continuous measurements that resemble today’s single crystal X-ray diffraction protocols.^3,16−18^

A third recent development concerns the methods for structure solution and refinement. Electron diffraction is affected by the problem that the diffracted beams have quite high intensities and can be diffracted multiple times within the same crystal, even if the crystal has a thickness of 10–30 nm only. This multiple scattering leads to a strong nonlinear modification of the reflection intensities, which hampers structure solution and refinement by classical methods. A simple approach to reduce the strength of multiple scattering is to use even thinner crystals or to combine multiple data sets.^19,20^ Alternatively, the multiple scattering can be considered in the structure refinement procedure, called dynamical refinement.^21−23^ Dynamical refinement has tremendously improved the reliability of the structure determination and allows even the detection of hydrogen atoms.^24^ Just recently, a further breakthrough was reported: Using dynamical refinement enables the determination of the absolute configuration, even much more reliable than by single-crystal X-ray diffraction.^25,26^

ED investigations are carried out in a transmission electron microscope (TEM). In the high vacuum inside the TEM, organic compounds tend to sublime. Furthermore, most organic compounds are rapidly destroyed by the electron beam. Both effects can be reduced by applying low temperatures, consequently, most 3D ED experiments on molecular crystals are performed at cryogenic temperatures (LT), typically around −180 °C.^2,27^ So far, 3D ED has only rarely been able to perform measurements on organics^28^ and MOFs^29^ at room temperature (RT). *In situ* investigations of phase transitions above room temperature with ED are challenging. Even inorganic materials are rarely investigated by ED at higher temperatures. One recent example is the investigation of the metal–insulator transition of VO_2_ crystals, studied at temperatures above 200 °C.^30^ To our knowledge, no profound temperature-dependent structural investigation above room temperature has been carried out on molecular crystals with electron diffraction so far. Here, we report on the crystal structure determination of the organic compound Pigment Orange 34 (C_34_H_28_Cl_2_N_8_O_2_) between −180 and +220 °C by high-end 3D ED methods.

**Figure 1:**
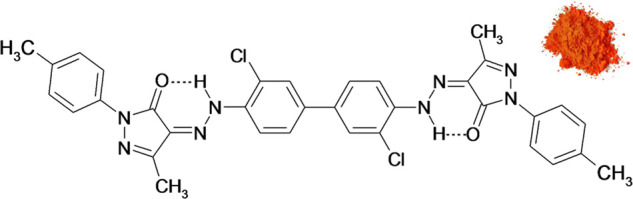
Chemical structure and color of Pigment Orange 34.

Pigment Orange 34 (P.O.34, Figure 1) is an industrial organic pigment used in printing inks, including textile printing, and for the coloration of plastics, especially PVC.^31^ Like other pigments, P.O.34 is fully insoluble in water and all solvents. In printing inks or plastics, the powder is not dissolved, but finely dispersed, and the resulting color depends on the crystal structure of the pigment. P.O.34 has been known since 1911^32−36^ and produced for more than 80 years. Although it is known that the optical properties of organic pigments strongly depend on their crystal structure (i.e., on the arrangement of molecules in the solid state), the crystal structure of P.O.34 has never been determined. Hitherto, only one crystal phase is known.

## Results and Discussion

Commercial samples of P.O.34 generally have very small crystallite sizes (about 30 nm), because smaller particles lead to a better transparency and to a higher color strength, i.e., a higher molar extinction, so that less pigment is required to achieve a given hue. In order to obtain a chemically pure compound, free of industrial additives, we synthesized P.O.34 by the usual route by bis-diazotization of 3,3′-dichlorobenzidine, followed by coupling with 5-methyl-2-*p*-tolyl-pyrazol-5-one in water (see Figure S4), resulting in a nanocrystalline powder (Figure S3). Numerous attempts were performed at recrystallization, e.g., from organic solvents at 150–300 °C. XRPD analysis of the resulting powders revealed that all samples exhibit the same polymorphic form as the industrial product. In some of the experiments, the crystallite size could be increased to about 90 nm, resulting in a powder pattern with sharper reflections than the industrial product (Figure S3).

**Figure 2:**
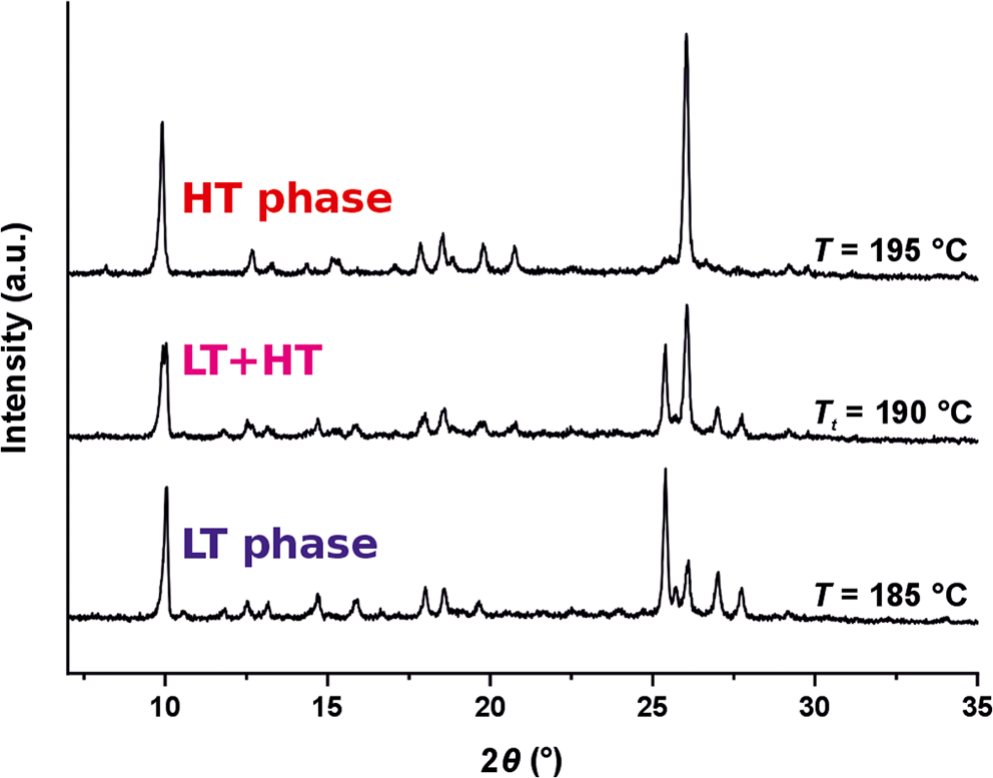
Temperature-dependent XRPD patterns of P.O.34 showing the phase transition from the LT phase to the HT phase upon heating (sample in capillary, Cu–K*α*_1_ radiation).

We searched for structural changes triggered by the temperature. Temperature-dependent XRPD revealed a first-order phase transition between 185 and 195 °C, yielding a new phase, which has never been described before (Figure 2, Figure S8). We call the known room-temperature phase the “LT phase”, and the new high-temperature phase “HT phase”. In the DSC analysis (Figure S6), the phase transition from the LT to the HT phase initiates at 180 °C. When P.O.34 is processed at temperatures above 180 °C in textile printing or plastics coloration, the HT phase is formed, with corresponding changes of the optical and mechanical properties.

The phase transition is reversible but with a considerable hysteresis: In the DSC, the back-transition from the HT to the LT phase starts at 140 °C without visibly losing crystallinity (Figure S7). P.O.34 melts under decomposition at 351 °C (Figure S6).

Reversible phase transitions are very rare in organic pigments. All commercial pigments form very dense, efficient molecular packings with high lattice energies and melting points far above 300 °C.^31^ The structures do not provide any free space for the molecules to move, which hampers any reversible structural changes. To the best of our knowledge, P.O.34 is the first instance of a reversible phase transition in an industrial organic pigment in the solid state. Since the phase transition is reversible, the HT phase cannot be obtained at RT. The quality of the powder patterns looked promising for structure solution from powder data, like it has been successfully done for many other organic pigments (see, e.g., refs (37−39)). However, we could not obtain a reliable index of the patterns. Thus, a classical structure solution from the powder data was not possible.

Therefore, we used 3D ED to determine the crystal structures of the LT and HT phases of P.O.34. A sample, which was recrystallized from boiling nitrobenzene (bp 211 °C) was dispersed in ethanol and sprayed on a film of amorphous carbon supported by a Cu TEM grid. Like most organic compounds, the crystals of P.O.34 were quite sensitive to the electron beam. To reduce the electron beam dose, one can measure at low temperatures and distribute the electron dose over a large area and thus completely illuminate a suitable large crystal.^5^ However, if the particles do not have a perfect morphology, tend to bend or even consist of several crystalline domains, the quality (reflection broadening, superposition of several single-crystal contributions, low resolution, etc.) of the diffraction patterns when a particle is fully illuminated will not be sufficient for a proper structural investigation.^25^ Another possibility is to use an electron beam much smaller than the lateral particle size for the electron diffraction experiments.

In this case, the illuminated area of the inhomogeneous particle is continuously changed during data acquisition.^12,40,41^ This reduces the chance of simultaneous illumination of several or strongly bent domains and also means that the electron dose can still be kept low in this way as it is distributed over the large area of the particle. We chose the latter approach, searched for thin crystals with a large lateral size of about 1 × 4 μm^2^ (Figure S9), and used a quasi-parallel electron beam with a diameter of about 200 nm. The data acquisition was performed in a sequential way using electron beam precession^42,43^ (PED). The tilt-dependent crystal movement was interpolated based on an own developed pretilt experiment done in STEM mode (Fast-ADT).^14^ Further details about the special techniques used in this work are given in the Supporting Information (Section 1).

**Figure 3:**
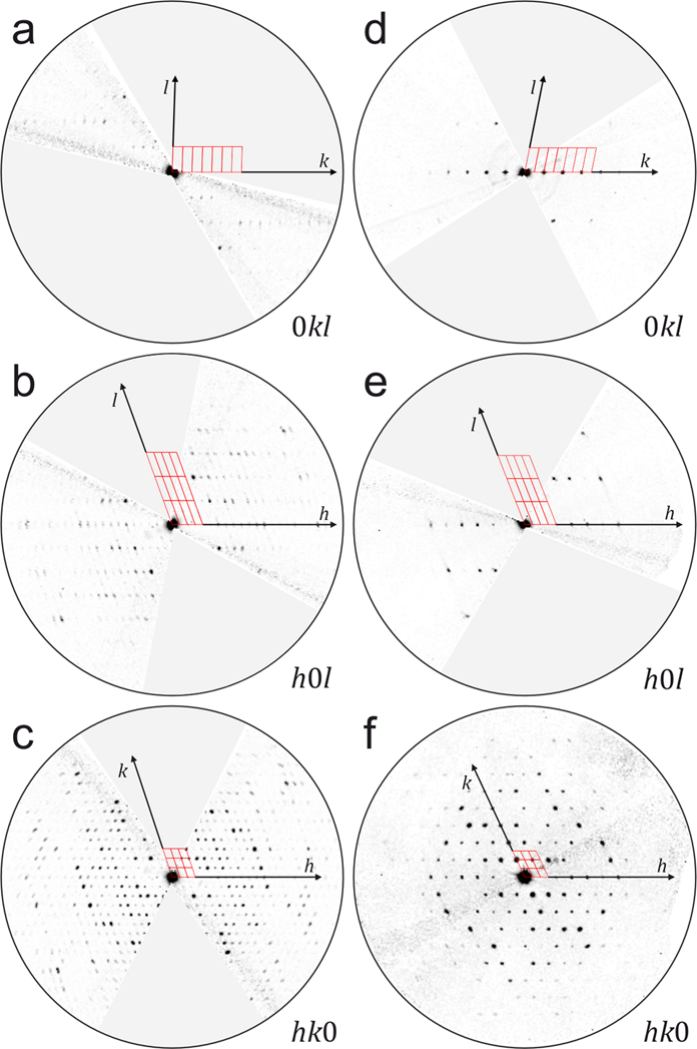
3D ED patterns of P.O.34, reconstructed from the individual measured frames. (a–c) LT phase, measured at −180 °C. (d–f) HT phase measured at 220 °C. For a better comparison of the two data sets, the directions of *h*, *k*, and *l* in the HT patterns correspond to the nonstandard *F*-centered unit-cell setting.

3D ED data of the LT phase were collected at RT and at −180 °C with accumulated electron doses of 45 e^–^/Å^2^ and 61 e^–^/Å^2^, respectively, distributed over large parts of the particle. For the determination of the crystal structure of the HT phase, 3D ED was performed at 220 °C by using a heating holder that allows tilting of the specimen at an angular range of approximately ±35°. Despite the high temperature and the high vacuum in the TEM, the crystals showed no tendency for sublimation. However, the beam sensitivity was much higher than at RT, and the crystals rapidly decomposed in the electron beam, although a very low electron beam dose of approximately 0.212 e^–^/Å^2^ s was used. Nevertheless, we could obtain reliable electron diffraction patterns from a couple of crystals (Figures 3, S10). The 3D ED patterns measured at RT and LT were quite similar, proving the absence of a phase transition between RT and LT. The patterns showed a triclinic unit cell with a volume of about 3000 Å^3^ (Table S1). According to Hofmann’s volume increments,^44^ the unit cell contains four molecules of P.O.34 per unit cell, which corresponds to 148 symmetrically independent atoms in space group *P*1̅. The HT phase has a triclinic unit cell, too, but its volume is only about 750 Å^3^, which corresponds to one molecule per unit cell.

The reflection integration of the LT and RT data sets resulted in merging error *R*_int_ of 17.4% and 12.2%, which are typical values for electron diffraction data. All attempts to solve the large structure of the LT phase with the usual methods, e.g. direct methods^45^ or charge flipping,^46^ failed. A closer look revealed that, especially in the LT data, the individual diffraction patterns showed deviating orientations and distortions, apparently caused by crystal bending and/or diffractive domains smaller than the particle size.^25^ To improve the reflection integration, the parameters describing the orientation of the individual diffraction patterns and their optical distortions caused by lens aberrations were optimized^47,48^ (Figure S13), drastically improving reflection integration (Figure S14) and therewith the *R*_int_ dropped to 7.9% for the LT and 8.5% for the RT data set. With these extracted data, it was possible to solve the structure from both individual data sets with SUPERFLIP,^49^ resulting in the expected 4 molecules per unit cell. All 92 non-hydrogen atoms could be clearly identified in the potential map (Figure S15). For the high-temperature 3D ED data recorded at 220 °C, we combined the data sets of three crystals, leading to better intensity statistics, reduced multiple scattering effects, and more accurate lattice parameters (Figure S14). The electron diffraction patterns showed reliable intensities of only up to 0.8 Å^–1^. The ED data allowed the crystal structure of the HT phase to be solved by Direct Methods^45^ in space group *P*1̅ (*Z* = 1). The structures of both phases (i.e., the LT, RT, and HT data sets) were refined with JANA2006^50^ using the kinematical approximation, converging to fairly good *R*_1_(obs)-values of 15.9%, 19.4%, and 12.8% respectively, for all observed reflections. The crystal structure of the LT phase (data sets measured at LT and RT) was additionally refined by a dynamical refinement, i.e., taking the multiple scattering into account. The refinements converged to very good *R*_1_(obs) values of 10.5% for the LT and 10.0% for the RT data set.

**Figure 4:**
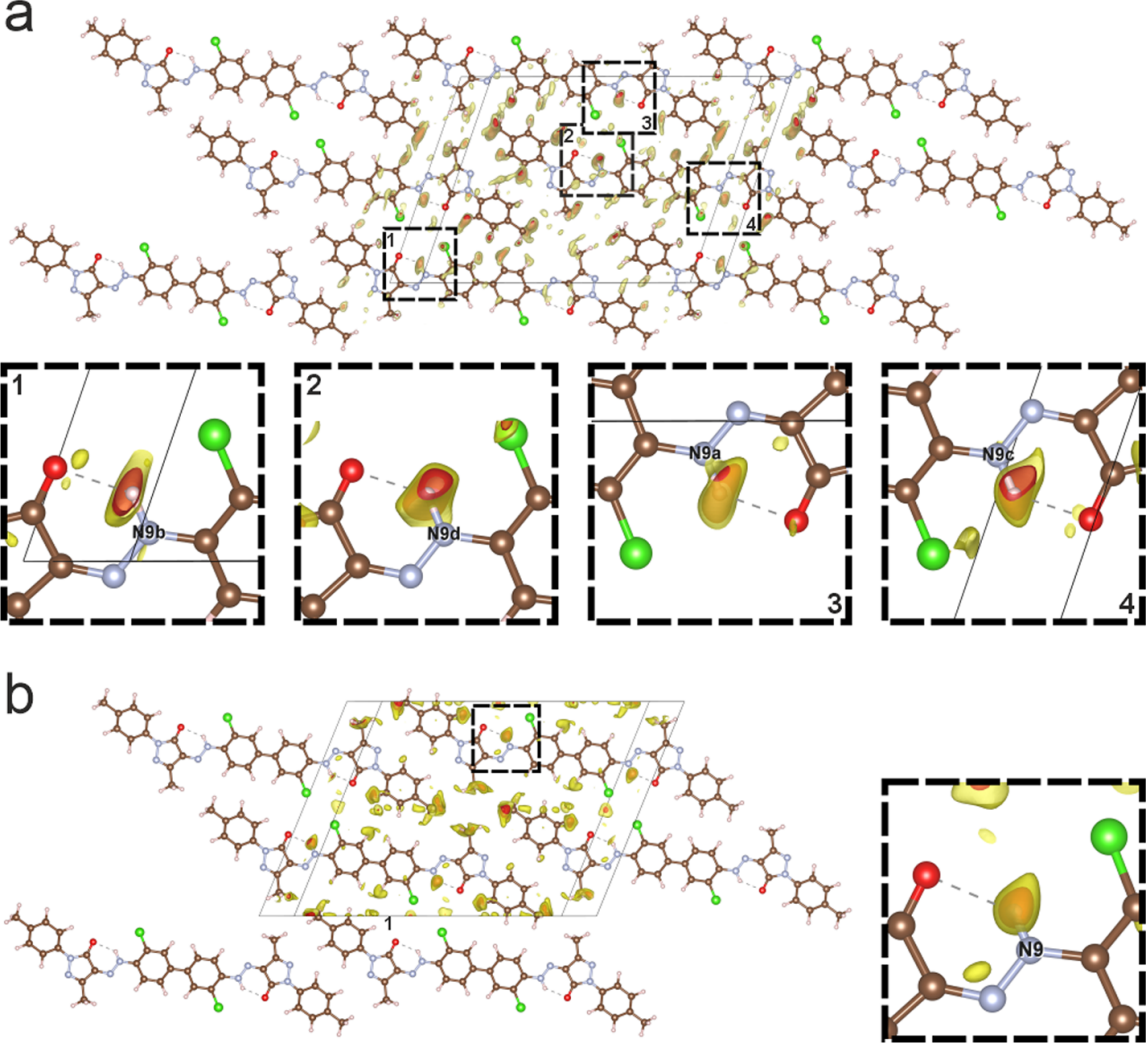
Localization of the H atoms of the hydrazone groups from difference Fourier maps obtained by 3D ED. (a) LT phase at −180 °C. Results from dynamical refinement, both symmetrically independent molecules shown. (b) HT phase, measured at 220 °C. The HT phase contains only one symmetrically independent molecule, situated on an inversion center. The red isosurfaces correspond to the 3σ[Δ*V*(***r***)] level. For further details, see Supporting Information.

The high quality of all data sets allowed us to locate even most of the hydrogen atoms in the difference Fourier maps in both phases, including the H atoms of all hydrazone groups. Thus, both phases were proven to exhibit the hydrazone-tautomeric form −NH–N=C instead of the azo form (−N=N–CH−) in the solid state (Figure 4). Further details about the data reconstruction and structure determination are given in the Supporting Information, Tables S2 and S3.^a^

**Figure 5:**
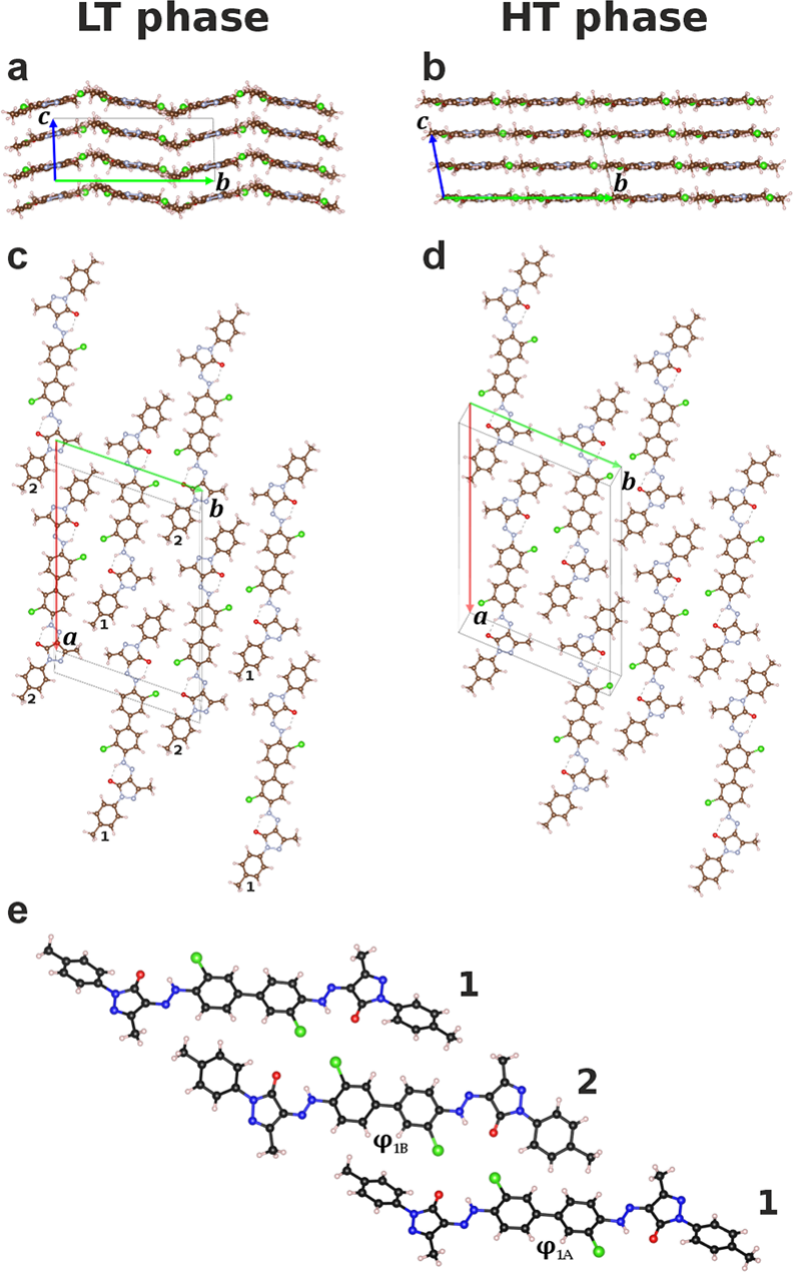
Crystal structures of P.O.34. (a, c, e) LT phase; (b, d) HT phase. (a, b) View along the ***a***-axis. (c,d) View perpendicular to the planes. 1 and 2 denote the two symmetrically independent molecules (e). For a better comparison of the two structures, the HT phase is presented in a nonstandard *F*-centered unit cell setting. Red, green and blue arrows (a, b, c, d) correspond to the ***a***, ***b***, ***c*** lattice vectors.

The crystal structures of both phases are shown in Figure 5. In the HT phase, the molecules are arranged in planar layers. The molecules are situated on crystallographic inversion centers. Correspondingly, the central biphenyl fragment is exactly planar. This planarity is a packing effect. An individual molecule would prefer a twisted conformation with a torsion angle ϕ_1_(Ph–Ph) of about 42°.

On the other hand, planar molecules allow a better molecular stacking in the crystal; correspondingly, most diaryl-hydrazone pigments adopt a planar or nearly planar conformation in the solid state.^51,31,11^ It is not visible if the biphenyl fragment is actually planar or if the two phenyl rings are librating around planarity. The pyrazole-hydrazone moiety is coplanar with the biphenyl fragment; only the terminal tolyl rings are slightly bent out of the plane by 9.7(12)°.

The unit cell of the LT phase contains two symmetrically independent molecules; both are located at general positions. The molecules differ in their conformation: In one molecule, the biphenyl fragment is almost planar (ϕ_1B_ = 179.3(3)°), in the other one it deviates by about 11.5(4)° from planarity (ϕ_1A_ = 168.5(4)°). The hydrazone moieties are coplanar to the biphenyl fragment, as usual for hydrazone pigments. The planarity of the chromophoric system explains the experimentally observed high color strength. The terminal tolyl groups are slightly twisted out of the molecular planes, with torsion angles between 155.4(7)° and 170.2(7)°. Similar values have also been found for monohydrazone-pyrazolone pigments.^52,53^ However, the terminal phenyl rings do not play a major role in the optical properties in these pigments.

Apparently, the LT phase is preferred at lower temperatures because it allows an energetically preferred twisting of the biphenyl fragment for half of the molecules, whereas the HT phase is preferred by entropy. Accordingly, the Debye–Waller factors, averaged over all non-hydrogen atoms, increase from 1.8 via 3.7 to 9.0 Å^2^ for the measurements at −180 °C, RT, and 220 °C, respectively. Also, the unit cell volume increases from 730 via 753 to 784 A^3^ per molecule.

The complicated structure of the LT phase with two symmetrically independent molecules and a large triclinic unit cell with four molecules was the reason indexing and structure solution from powder data failed. However, Rietveld refinements were possible. For this task, XRPD data were collected at −180, RT, and 220 °C. Starting from the crystal structures determined by 3D ED, Rietveld refinements were performed with the program TOPAS.^54^ The refinements converged with low *R* values and good fits for the LT phase at −180 °C and RT (Figure S18, Figure S19, and Table S4). Despite good agreement in principle in the case of the Rietveld refinement of HT phase, the experimental and simulated profiles show differences. This is probably due to stacking disorder and/or an incomplete phase transition. The structures refined from powder data are very similar to those of 3D ED: the root mean-square Cartesian deviation (RMSCD)^55^ of the coordinates of all atoms (except H atoms) are 0.013, 0.015, and 0.025 Å for both LT phases and the HT phase.

The crystal structure of both phases were confirmed by lattice-energy minimization with dispersion-corrected DFT (DFT-D) calculations with the program GRACE,^55^ using a Perdew–Burke–Ernzerhof (PBE) functional^56^ and a dispersion correction by Grimme.^57^ Upon full optimization of lattice parameters and atomic coordinates, the crystal structures of both phases determined by 3D ED were perfectly reproduced (Figures S20, S21): RMSCD of the coordinates of all atoms (except H atoms) was as low as 0.049 Å for the LT phase and 0.073 Å for the HT phase. The LT phase was proven to have a more favorable lattice energy, with a calculated enthalpy difference of 5.8 kJ/mol.

The color of organic pigments depends on the molecular conformation and on the packing of the molecules.^31^ Correspondingly, polymorphs frequently differ in their colors (Color polymorphism).^58^ During industrial processing for coloring plastics or textile printing, temperatures of more than 180 °C can be reached, which in the case of P.O.34 leads to a phase transition from the LT to the HT phase. In order to investigate the influence of temperature and phase transition on the optical properties, temperature-dependent UV–vis measurements were carried out. At HT, the band of the UV–vis reflectance spectrum shifts significantly from 600 nm (RT) to 616 nm (Figure S22). However, upon cooling to RT, the original optical properties of P.O.34 are restored. To observe the effects of the phase transition or structural change on the optical properties independently of the influence of temperature, heating to 220 °C and subsequent cooling to RT in 10 °C steps was recorded with UV–vis. Interestingly, no discontinuous changes in the spectra are observed during both heating and cooling. Accordingly, the phase transition hysteresis LT ↔ HT cannot be observed in the spectral range of UV–vis measurements. Consequently, the phase transition itself seems to have little influence on the optical properties. Instead, a reversible and continuous linear change of the band at 616 nm as a function of temperature is visible (Figure S23). Correspondingly, P.O.34 can, in principle, be used as an optical temperature sensor.

## Conclusion

The present work shows that crystal structures of organic compounds can be reliably determined by 3D electron diffraction, even for medium-sized molecules. The RT phase of P.O.34, containing 148 symmetrically independent atoms, is one of the largest unknown organic structures that has ever been solved by using only the electron diffraction data. Even the hydrogen atoms of the hydrazone groups could be localized by difference Fourier synthesis. Usually, the structural investigation of organic compounds using ED ends here at the latest. We went a step further and dared to investigate the HT phase of P.O.34 at 220 °C by electron crystallography. To our knowledge, the HT phase is the first *in situ* crystal structure determination at HT of an organic compound using ED, made possible by the specially developed and explained measurement methodologies presented here. Despite the high temperature, hydrogen atoms could be localized. We are confident that 3D ED will be an important investigation method for *in situ* studies of fine crystalline organic compounds, MOFs and COFs, even at high temperatures.

## Supporting Information Available

Details on experimental section, details on three-dimensional electron diffraction, synthesis, NMR, Δ*T*-XRPD, thermal analysis, UV–vis, TEM images and crystallographic details, Structure refinement (PDF)

## Footnotes

a Pigment Orange 34: Orange powder, C_34_H_28_Cl_2_N_8_O_2_, M_r_ = 651.6 g/mol. (a) Low-temperature phase (LT phase), determined at 97 K: crystal size 0.004 × 0.001 × 0.00005 mm^3^, triclinic, *P*1, *a* = 25.101(9) Å, *b* = 18.238(7) Å, *c* = 7.232(4) Å, α = 84.62(3)°, β = 69.21(3)°, γ = 70.87(3)°, *V* = 2923(2) Å^3^, *Z* = 4, ρ_calc_ = 1.483 g·cm^–3^, electron radiation, λ = 0.01970 Å, resolution 1.0 Å^–1^, dynamical refinement, No. of used reflections (obs/all) 3992/13699, 395 parameters, *R*_1_(obs) = 10.5%, *wR*_1_(all) = 12.1%, *GoF*(all) = 1.53 (b) LT phase, determined at 293 K: crystal size 0.004 × 0.001 × 0.00009 mm^3^, triclinic, *P*1, *a* = 25.129(6) Å, *b* = 18.45(1) Å, *c* = 7.358(2) Å, α = 84.98(3)°, β = 69.22(2)°, γ = 70.89(3)°, *V* = 3012(2) Å^3^, *Z* = 4, ρ_calc_ = 1.439 g·cm^–3^, electron radiation, λ = 0.01970 Å, resolution 1.2 Å^–1^, dynamical refinement, No. of used reflections (obs/all) 4667/18281, 306 parameters, *R*_1_(obs) = 10.0%, *wR*_1_(all) = 11.9%, *GoF*(all) = 1.48. (b) High-temperature phase (HT phase), determined at 493 K: crystal size 0.0010 × 0.0001 × ∼0.00005 mm^3^, triclinic, *P*1, *a* = 11.915(4) Å, *b* = 10.348(4) Å, *c* = 7.394(4) Å, α = 108.34(4)°, β = 88.30(3)°, γ = 114.21(3)°, *V* = 784.1(7) Å^3^, *Z* = 1 (respectively *F*1, *a* = 25.16(1) Å, *b* = 19.666(7) Å, *c* = 7.400(7) Å, α = 92.60(5)°, β = 71.26(5)°, γ = 67.35(3)°, *V* = 3140(4) Å^3^, *Z* = 4), ρ_calc_ = 1.382 g·cm^–3^, electron radiation, λ = 0.01970 Å, resolution 1.0 Å^–1^, kinematical refinement, No. of used reflections (obs/all) 368/1147, 80 parameters, *R*_1_(obs) = 12.8%, *wR*_1_(all) = 26.6%, *GoF*(all) = 3.06. CCDC 2314856, 2314857, and 2314855 contain the supplementary crystallographic data of the structures determined at 97, 293, and 493 K.
