# Supplementary material for: High Temperature Electron Diffraction on Organic Crystals: In Situ Crystal Structure Determination of Pigment Orange 34
Source: J Am Chem Soc. 2024 Mar 27;146(14):9880–7. doi: 10.1021/jacs.3c14800 (PMC11009952; doi:10.1021/jacs.3c14800)
Supplement: Supplementary file 1 — ja3c14800_si_001.pdf [file ja3c14800_si_001.pdf]

Supporting Information

# High Temperature Electron Diffraction on Organic Crystals: *in situ* Crystal Structure Determination of Pigment Orange 34.

Yaşar Krysiak,*[a] Sergi Plana-Ruiz,[b, c] Lothar Fink,[d] Edith Alig,[d] Ulrich Bahnmüller,[a] Ute Kolb,[b, e] and Martin U. Schmidt*[d]

# Table of Contents

1 Explanations ... 2
1.1 Three-dimensional electron diffraction (3D ED) ... 2
1.1.1 Introduction ... 2
1.1.2 Acquisition methods ... 3
1.1.3 Our approach ... 4
2 Experimental Procedures ... 6
2.1 Synthesis of Pigment Orange 34 ... 6
2.2 Recrystallisation of Pigment Orange 34 in nitrobenzene ... 6
2.3 Differential thermal analysis/calorimetry and thermogravimetry ... 6
2.4 Ultraviolet-visible (UV-Vis) spectroscopy ... 6
2.5 3D electron diffraction ... 7
2.5.1 Measurements ... 7
2.5.2 Data reconstruction ... 7
2.6 Powder X-ray diffraction ... 7
2.6.1 Experimental setup ... 7
2.6.2 Temperature dependent measurements ... 8
2.6.3 Rietveld refinement ... 8
3 References ... 8
4 Figures and Tables ... 10
5 Crystal structure data of P.O.34 determined by Rietveld refinement based on PXRD data recorded at 93, 293 and 493 K. ... 25

# 1 Explanations

## 1.1 Three-dimensional electron diffraction (3D ED)

### 1.1.1 Introduction

Three-dimensional electron diffraction (3D ED) is an experimental technique based on the reconstruction of the observable diffraction space by means of electron diffraction patterns, regardless of the acquisition method used to collect the data and the degree of crystallinity of the material under study.[1] The idea is to use an electron beam to acquire several diffraction patterns from an individual particle in different geometric orientations, usually following an approach similar to that of a tomography experiment.[2,3] However, the main difference between image tomography and 3D ED is that images in the former case consist of projections of the object to be reconstructed in real space, while in the latter case diffraction patterns directly correspond to sections of the observable diffraction space from the illuminated object.

The reconstruction of the collected patterns into the 3D space and its subsequent and careful analysis of the diffracted intensity distribution allows the crystallographic characterisation of a wide range of materials, e.g., from organics to disordered zeolites or metal organic frameworks.[4–7] In case of

crystalline solids, their unit cell can be determined and their crystal structure can be solved and further refined to a high level of accuracy.[8–11] In this context, the use of a transmission electron microscope (TEM) can be considered as an electron nano-diffractometer since the acquisition and processing of the diffraction data is similar to single-crystal X-ray diffraction methods. The major advantage of electrons with respect to X-rays is that they can be focused down to the scale of picometres, thus tiny nanometre-sized domains can be individually probed by an electron beam, which is not achievable by X-rays. On the other hand, the stronger interaction of electrons with matter means that the interpretation of reflection intensities with the kinematical theory of diffraction is not likely to hold, and multiple scattering events need to be considered to properly analyse them. Nevertheless, dynamical diffraction effects can be currently considered by using the routinely used *Jana2006*/*Dyngo* program for crystal structure refinement. Therefore, it is possible to obtain an accurate and reliable structural model based solely on electron diffraction data.

### 1.1.2 Acquisition methods

There are several methods to acquire 3D ED data that are differentiated according to how the diffraction space is sampled. The initial technique introduced by the group of Ute Kolb in 2007[2] was based on the acquisition of diffraction patterns at tilt steps of 0.5°-1°, while acquiring TEM or STEM images prior to the pattern collection to assert that the electron beam was illuminating the targeted crystal. A procedure firstly coined automated diffraction tomography (ADT), but also called electron diffraction tomography (EDT) in other works.[12] Although the crystallographic results were very impressive at that time, a lot of potential data was not recorded in-between the diffraction patterns (the missing step wedges), which triggered the development of other routines to minimize this loss. The first instance is the coupling of the initial ADT technique with a precessed electron beam, the so-called precession electron diffraction tomography (PEDT).[4] In this situation, the precession of the beam (usually set between 0.5° and 1.5°) induces the Ewald sphere to sweep the missing step wedge and integrate the reflection intensities on the detector, hence increasing the probability to measure them at their maxima and minimize the dynamical effects.[13] Averaged information of the missing step wedge is retrieved by using the same number of tilt steps as ADT. An alternative acquisition method to PEDT is the rotation electron diffraction (RED) technique.[14] RED performs the tilt of the crystal during the 3D ED acquisition in two steps; fine beam tilt steps of 0.01°-0.2° combined with coarse goniometer tilt steps of 2°-4°. The fine slicing of the diffraction space results in finer rocking curves that allow to precisely determine the maximum intensity or integrate (software-wise) each reflection. Although the approach becomes better for unit cell determination, dynamical effects such as Kikuchi lines, double-diffraction or intensity redistributions cannot be minimized as in PEDT, thus complicating the crystal structure determination. Finally, a last approach is the integrated electron diffraction tomography (IEDT)[15,16], which in some other works is referenced as MicroED[17] or continuous RED.[15,18] IEDT performs the acquisition while the sample is continuously tilted, resulting in a slightly reduced electron dose on the crystal, an integration of the reflection intensities on the detector as well as, in some cases, a fine slicing of the observable diffraction space. Also, IEDT does not apply a beam shift during the acquisition, thus generally relying on the crystal staying in the illuminated area. PED and IEDT techniques can be combined in order to integrate reflections that are not covered when the camera is in stand-by (the time when the electronics are reading the data from the chip and saving it to the disk, i.e., the read-out time). If stage velocity and precession angle are properly set, the sampled diffraction space can be maximized.[16] See Figure S1 for a schematic representation of the diffraction space sampling according to the different acquisition methods.

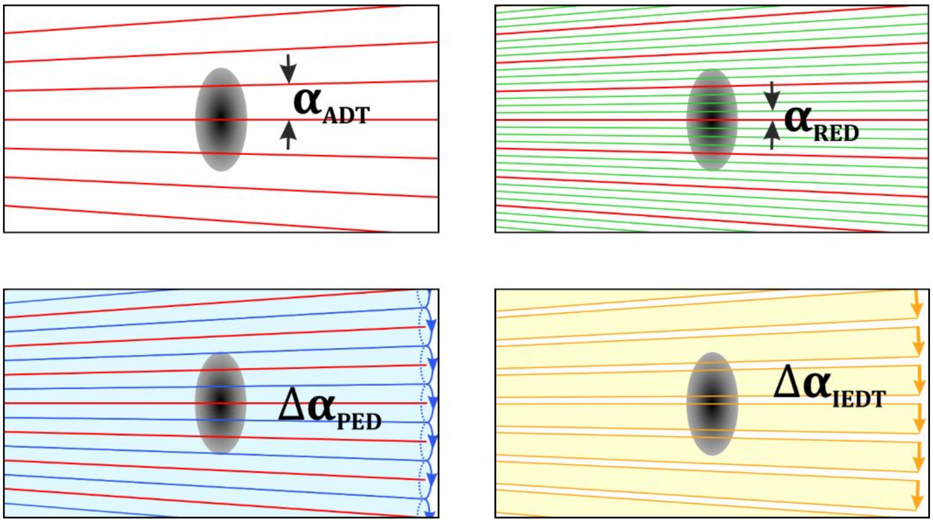


*Figure S1. The different ways to sample one reflection in the diffraction space according to the developed 3D ED acquisition methods; Automated Diffraction Tomography (ADT), Rotation Electron Diffraction (RED), Precession Electron Diffraction Tomography (PEDT or simply ADT in some works) and Integrated Electron Diffraction Tomography (IEDT or MicroED in other works). Red stands for mechanical and sequential stage tilt, green for beam tilt, blue for electron beam precession movement and orange/yellow for continuous tilt of the stage.*

### 1.1.3 Our approach

In this work, PED patterns were acquired instead of continuously tilting the crystal during the data collection. This option was chosen because a continuous tilt requires a beam illumination that shines large parts of the crystal under study but also a lot of background, even if a high mechanically-stable stage is used. Although that may not be a problem for well crystalline micro-sized crystals, the reality is that most submicron crystals are slightly bended, composed of misoriented domains and have thickness variations across the particle. This results in the smearing of the reflection intensities and complicates the final refinement steps of the crystal structure. For these reasons, a small beam of around 200 nm was set to illuminate thin parts of a bigger crystal and get diffraction patterns from a few domains. This is achievable in a TEM by using specific lenses settings and a small aperture (usually 10 µm) in the condenser system, commonly referred as nano-beam electron diffraction (NBED) mode. NBED is also advantageous because of the interaction of the electron probe onto the targeted crystal without illuminating other particles, a characteristic not possible to obtain when using selected-area electron diffraction (SAED), the frequent illumination mode of continuous-tilting approaches. SAED relies on an aperture in the projector system of the TEM and a well-spread beam on an area bigger than the feature of interest (see Figure S2). The SAED aperture allows to select the illuminated region that will result in the diffraction pattern, but does not avoid the over-exposure of the electron beam to other near-by crystals, hence being detrimental to the measurement of beam-sensitive materials.

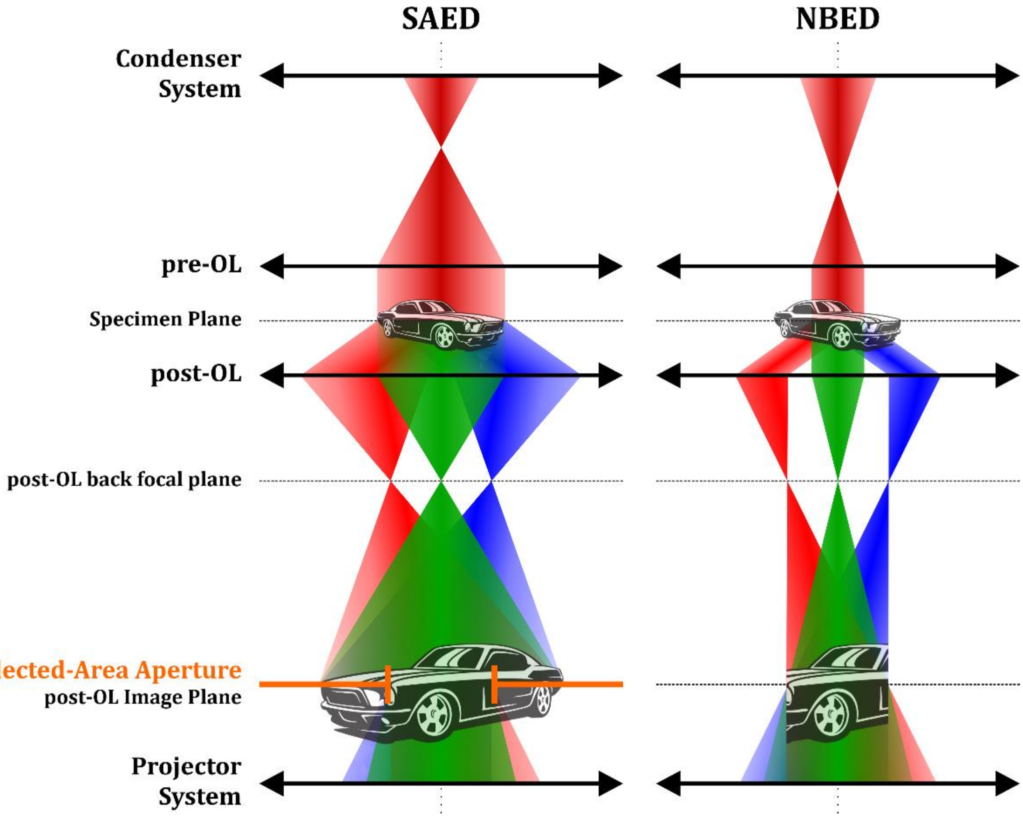


*Figure S2. Schematic representation of the optical path of the electron beam when acquiring diffraction data in selected-area electron diffraction (SAED) mode in comparison to nano-beam electron diffraction (NBED) mode.*

Another important point to bear in mind is the automation of the 3D ED data acquisition, especially for materials that lose their crystallinity under the electron beam. Automation is key for these compounds because the exposure of candidate crystals to the electron beam is only applied when necessary, resulting in larger 3D ED datasets of high-resolution diffraction patterns. In this work, the diffraction data of P.O.34 was collected with the Fast-ADT approach that uses the NBED illumination mode.[19] This acquisition method is based on two consecutive tilt scans of the stage along the angular range of the 3D ED experiment; the first one acquires (S)TEM images of the targeted crystal at different tilt angles to digitally select the position of the electron probe, and the second one is the acquisition of the PED patterns while the beam is physically shifted to track the crystal according to the pre-defined positions. Such approach reduces the experimental acquisition time and the collection of 3D ED data becomes a more routine and systematic technique. Moreover, the possibility to accurately define the electron beam positions for the entire 3D ED acquisition allows the shift of the beam along an area of the targeted crystal, which ensures that only a few domains are illuminated and they are not exactly the same for each pattern, so that the total beam damage on the crystal under study is minimized. As a further advantage, the digital pre-selection of beam positions offers the chance to acquire more than one 3D ED dataset during a single tilt scan; one dataset for each pre-selected crystal. In other words, to acquire one diffraction pattern from each pre-defined crystal location for every tilt angle of the chosen angular range of the 3D ED experiment. Ultimately increasing the throughput of high-quality electron diffraction data, even from beam sensitive materials.

# 2 Experimental Procedures

## 2.1 Synthesis of Pigment Orange 34

3.26 g (10.0 mmol) of 3,3'-dichlorobenzidine dihydrochloride were added to 33 mL of $HCl_{aq}$ (5.0 M) and 75 mL of purified $H_2O$. After stirring for 1 h a very finely dispersed suspension is obtained. The suspension was cooled to 0°C by direct addition of ice and diluted to 500 mL. In addition, cooling was carried out with an ice bath. 4.5 mL (22.5 mmol, 2.25 eq.) $NaNO_2$ (aq) (5.0 M) was injected to the suspension and stirred at 0°C for 1 h. To the resulting solution, 2.40 g (24.7 mmol, 2.5 eq.) of amidosulfonic acid was added and stirred for 1 h at 0°C. The reaction was tested for nitrite with KI-starch paper and if positive, some more amidosulfonic acid was added. Under gentle heating to 40°C, 25.0 mmol (2.5 eq.) of the corresponding pyrazolone compound was completely dissolved in 12 mL $HCl_{aq}$ (5.0 M) and 43 mL purified $H_2O$ in a separate flask and then transferred to a dropping funnel. To the bisdiazonium solution, 1 mL of pyridine was added as catalyst. The pyrazolone solution was then added to the bisdiazonium solution within 10 minutes and subsequently stirred for 2 hours. The resulting pigment suspension is heated to 55°C.

To check for remaining diazonium compounds, a small sample of the suspension was spotted onto a paper strip soaked in potassium carbonate-alkaline H-acid solution (4-amino-5-hydroxynaphthalene-2,7-disulfonic acid). If a blue colouration is visible on the soaked paper strip, a small amount of the corresponding pyrazolone compound was added to the pigment suspension and the test was repeated after 5 minutes. If there was no blue colouration of the soaked paper strip, the pigment suspension was stirred at 95 °C for 1 hour. After cooling to room temperature, the product was filtered and wash with 2 L of purified $H_2O$ until chloride free and pH neutral. The solid was dried at 80°C for at least 2 days. The yield is about 95%. The reaction scheme is shown in Figure S4. The corresponding $^1$H NMR of the product is shown in Figure S5.

## 2.2 Recrystallisation of Pigment Orange 34 in nitrobenzene

100 mg of P.O.34 was added to 10 mL nitrobenzene. The mixture was then boiled under reflux and solvent was added in steps of 5 mL until the pigment was completely dissolved or a total volume of 60 mL was reached. After complete solvation, the solution was heated to boiling for a further 5 minutes under reflux. If, on the other hand, the pigment was not completely dissolved, this period was extended to 2 hours. The magnetic stirrer core was then removed and the sample allowed to cool to room temperature. The crystals were collected by paper filtration. The sample was then dried at room temperature.

## 2.3 Differential thermal analysis/calorimetry and thermogravimetry

The thermal decomposition reactions were performed in a nitrogen atmosphere (purity: 5.0) in $Al_2O_3$ crucibles using a TG-DTA 92 thermo balance from SETARAM. The instrument was calibrated using standard reference materials. The measurement was performed with a heating rate of 3 K $min^{-1}$ and a flow rate of 75 mL $min^{-1}$. The results were confirmed by a differential thermal calorimetry (DSC). DSC measurements were carried out with a SETARAM DSC 131 device in a nitrogen atmosphere with a heating rate of 4.3 K $min^{-1}$ from room temperature up to 250 °C and then cooling down to RT with the same cooling rate. For each measurement about 10-15 mg of the sample was placed in a corundum crucible.

## 2.4 Ultraviolet-visible (UV-Vis) spectroscopy

Temperature dependent UV-Vis measurements of P.O.34 (mixed with $BaSO_4$) were acquired with an Agilent 8453 Carry 4000 with a praying mantis unit using a heating cell. A constant flow of nitrogen was passed through the heating cell. Measurements at room temperature and 220 °C were also

performed with pure $BaSO_4$ for baseline correction. The sample was first heated to 220 °C in order to remove volatile impurities. For heating up and cooling down experiments a measurement was taken 2 minutes after the desired temperature was reached.

## 2.5 3D electron diffraction

### 2.5.1 Measurements

All measurements of P.O.34 were carried out with a transmission electron microscope (TEM) FEI Tecnai F30 S-TWIN equipped with a field emission gun (300 kV) at the University of Mainz. The powdered samples were dispersed in ethanol using an ultrasonic bath and sprayed on carbon-coated copper grid using an ultra sound sonifier.[4] TEM images and electron diffraction (ED) patterns were acquired with a Gatan UltraScan4000 CCD camera (16-bit, 4096 x 4096 pixel) at hardware-binning of 2. Scanning transmission electron microscopy (STEM) images were collected by a FISCHIONE high-angular annular dark field (HAADF) detector and acquired by Emispec ES Vision software. A condenser aperture of 10 µm and mild illumination settings (gun lens 8, spot size 8) were used in order to produce a semi-parallel beam of 200 nm in diameter (0.212 e-/Å$^2$s). Crystal position tracking was performed in microprobe STEM mode. The ED data were collected with electron beam precession (precession electron diffraction, PED) to improve reflection intensity integration quality.[4,13] PED was performed using a Digistar unit developed by NanoMEGAS SPRL. The precession angle was kept at 1.0°. Cooling experiments were performed with a Gatan cryo-transfer tomography holder, whereas the heating experiments were realised with YYY. Three-dimensional electron diffraction data were collected using the acquisition module fast and automated electron diffraction tomography (Fast-ADT) developed for FEI and JEOL microscopes.[19]

### 2.5.2 Data reconstruction

PETS2.0 were used for 3D electron diffraction data processing.[20] It was necessary to carefully refine the orientation of each single frame due to bending and/or multiple domains of the measured particle to improve the data integration from $R_{int}$(all) = 17.4 % (LT) / 12.2 % (RT) down to 7.9 % / 8.5 % (Laue class -1). The HT datasets were merged. For this purpose, a reference data set and thus a reference orientation matrix was defined. In the next step, the position angles of the other data sets were transformed to match the reference orientation matrix. In a final step, the orientation and distortion of each frame had to be carefully refined for the respective merged datasets before the intensities were extracted.

#### *High temperature phase*

First, each dataset was processed separately to determine its individual orientation matrix. Then, a reference orientation matrix was defined and the orientation angles of the other datasets were transformed to match the reference one. In a final step, the orientation and distortions of each individual pattern were carefully refined before the intensities were extracted. The reflection integration (645 observed of 2218 reflections) of the merged HT dataset resulted in a merging error $R_{sym}$ of 8.4 % for the estimated Laue class -1 (377 observed of 1156 observed reflections). The noise for the last two averaged rocking-curves of the reflections (Figure S17) indicate that the data only have reliable intensities up to about 0.8 Å$^{-1}$.

## 2.6 Powder X-ray diffraction

### 2.6.1 Experimental setup

Powder diffraction was performed on a STOE transmission powder diffraction system (STADI P) in Debye-Scherrer geometry equipped with a Ge(111) monochromator (Cu-K$\alpha_1$ radiation, $\lambda$ = 154.056 pm) and a linear position-sensitive detector. The sample was contained in a glass capillary with 0.7

mm diameter, which was spun during the measurement. The measurement covered the range from 2° to 80° in 2$\vartheta$ with a step width of 0.01°. The software WinXPow[21] was used for data acquisition.

### 2.6.2 Temperature dependent measurements

The sample was heated up to 220 °C on the powder diffractometer with a cryostream unit. The heating rate was 6 K/min. The measurement programme is shown in Figure S8b. Powder pattern were measured in the 2θ range 2-38° with a PSD-step of 1.5° at 30 s/step every 50 °C in the range from 20 °C to 150 °C. From 150 °C onwards, a measurement was carried out every 5 °C with the same parameters. At 190 °C a phase transformation could be observed. The powder diagrams are shown in Figure S8a. At 220 °C the temperature was kept constant and a long-term measurement was carried out.

### 2.6.3 Rietveld refinement

The Rietveld refinements were carried out with TOPAS[22] Version 6. The bond distances and angles of the molecules were restrained to the structural motifs found in the CSD database. The isotropic displacement parameters of the carbon, nitrogen and oxygen atoms were kept the same, while the parameters of the chlorine and hydrogen atoms were constrained to be 1.2 resp. 1.5 times of the C, N and O atoms.

# 3 References


(1) Gemmi, M.; Mugnaioli, E.; Gorelik, T. E.; Kolb, U.; Palatinus, L.; Boullay, P.; Hovmöller, S.; Abrahams, J. P. 3D Electron Diffraction: The Nanocrystallography Revolution. *ACS Cent. Sci.* **2019**, *5* (8), 1315–1329. https://doi.org/10.1021/acscentsci.9b00394.

(2) Kolb, U.; Gorelik, T.; Kübel, C.; Otten, M. T.; Hubert, D. Towards Automated Diffraction Tomography: Part I - Data Acquisition. *Ultramicroscopy* **2007**, *107* (6–7), 507–513. https://doi.org/10.1016/j.ultramic.2006.10.007.

(3) Kolb, U.; Krysiak, Y.; Plana-Ruiz, S. Automated Electron Diffraction Tomography – Development and Applications. *Acta Cryst B* **2019**, *75* (4), 463–474. https://doi.org/10.1107/S2052520619006711.

(4) Mugnaioli, E.; Gorelik, T.; Kolb, U. Ab-Initio Structure Solution from Electron Diffraction Data Obtained by a Combination of Automated Diffraction Tomography and Precession Technique. *Ultramicroscopy* **2009**, *109* (6), 758–765. https://doi.org/10.1016/j.ultramic.2009.01.011.

(5) Mugnaioli, E.; Kolb, U. Structure Characterization of Nanocrystalline Porous Materials by Tomographic Electron Diffraction. *Z Krist-Cryst Mater* **2015**, *230* (4), 271–288. https://doi.org/10.1515/zkri-2014-1805.

(6) Bruhn, J. F.; Scapin, G.; Cheng, A.; Mercado, B. Q.; Waterman, D. G.; Ganesh, T.; Dallakyan, S.; Read, B. N.; Nieusma, T.; Lucier, K. W.; Mayer, M. L.; Chiang, N. J.; Poweleit, N.; McGilvray, P. T.; Wilson, T. S.; Mashore, M.; Hennessy, C.; Thomson, S.; Wang, B.; Potter, C. S.; Carragher, B. Small Molecule Microcrystal Electron Diffraction for the Pharmaceutical Industry–Lessons Learned From Examining Over Fifty Samples. *Frontiers in Molecular Biosciences* **2021**, *8*.

(7) Andrusenko, I.; Gemmi, M. 3D Electron Diffraction for Structure Determination of Small-Molecule Nanocrystals: A Possible Breakthrough for the Pharmaceutical Industry. *WIREs Nanomedicine and Nanobiotechnology* **2022**, *14* (5), e1810. https://doi.org/10.1002/wnan.1810.

(8) Palatinus, L.; Petříček, V.; Corrêa, C. A. Structure Refinement Using Precession Electron Diffraction Tomography and Dynamical Diffraction: Theory and Implementation. *Acta Cryst A, Acta Cryst Sect A, Acta Crystallogr A, Acta Crystallogr Sect A, Acta Crystallogr A Cryst Phys Diffr Theor Gen Crystallogr, Acta Crystallogr Sect A Cryst Phys Diffr Theor Gen Crystallogr* **2015**, *71* (2), 235–244. https://doi.org/10.1107/S2053273315001266.

(9) Palatinus, L.; Corrêa, C. A.; Steciuk, G.; Jacob, D.; Roussel, P.; Boullay, P.; Klementová, M.; Gemmi, M.; Kopeček, J.; Domeneghetti, M. C.; Cámara, F.; Petříček, V. Structure Refinement Using Precession Electron Diffraction Tomography and Dynamical Diffraction: Tests on Experimental Data. *Acta Cryst B* **2015**, *71* (6), 740–751. https://doi.org/10.1107/S2052520615017023.

(10) Bowden, D.; Krysiak, Y.; Palatinus, L.; Tsivoulas, D.; Plana-Ruiz, S.; Sarakinou, E.; Kolb, U.; Stewart, D.; Preuss, M. A High-Strength Silicide Phase in a Stainless Steel Alloy Designed for Wear-Resistant Applications. *Nature Communications* **2018**, *9* (1), 1–10. https://doi.org/10.1038/s41467-018-03875-9.

(11) Fillafer, N.; Kuper, H.; Schaate, A.; Locmelis, S.; Becker, J. A.; Krysiak, Y.; Polarz, S. Design of Active Defects in Semiconductors: 3D Electron Diffraction Revealed Novel Organometallic Lead Bromide Phases Containing Ferrocene as Redox Switches. *Advanced Functional Materials* **2022**, *n/a* (n/a), 2201126. https://doi.org/10.1002/adfm.202201126.

(12) Palatinus, L.; Jacob, D.; Cuvillier, P.; Klementová, M.; Sinkler, W.; Marks, L. D. Structure Refinement from Precession Electron Diffraction Data. *Acta Cryst A, Acta Cryst Sect A, Acta Crystallogr A, Acta Crystallogr Sect A, Acta Crystallogr A Found Crystallogr, Acta Crystallogr Sect A Found Crystallogr* **2013**, *69* (2), 171–188. https://doi.org/10.1107/S010876731204946X.

(13) Vincent, R.; Midgley, P. A. Double Conical Beam-Rocking System for Measurement of Integrated Electron Diffraction Intensities. *Ultramicroscopy* **1994**, *53* (3), 271–282. https://doi.org/10.1016/0304-3991(94)90039-6.

(14) Zhang, D.; Oleynikov, P.; Hovmöller, S.; Zou, X. Collecting 3D Electron Diffraction Data by the Rotation Method. *Zeitschrift für Kristallographie* **2010**, *225* (2–3). https://doi.org/10.1524/zkri.2010.1202.

(15) Nederlof, I.; van Genderen, E.; Li, Y.-W.; Abrahams, J. P. A Medipix Quantum Area Detector Allows Rotation Electron Diffraction Data Collection from Submicrometre Three-Dimensional Protein Crystals. *Acta Cryst D* **2013**, *69* (7), 1223–1230. https://doi.org/10.1107/S0907444913009700.

(16) Gemmi, M.; La Placa, M. G. I.; Galanis, A. S.; Rauch, E. F.; Nicolopoulos, S. Fast Electron Diffraction Tomography. *J Appl Cryst, J Appl Crystallogr* **2015**, *48* (3), 718–727. https://doi.org/10.1107/S1600576715004604.

(17) Nannenga, B. L.; Shi, D.; Leslie, A. G. W.; Gonen, T. High-Resolution Structure Determination by Continuous-Rotation Data Collection in MicroED. *Nature Methods* **2014**, *11* (9), 927–930. https://doi.org/10.1038/nmeth.3043.

(18) Wang, Y.; Takki, S.; Cheung, O.; Xu, H.; Wan, W.; Öhrström, L.; Ken Inge, A. Elucidation of the Elusive Structure and Formula of the Active Pharmaceutical Ingredient Bismuth Subgallate by Continuous Rotation Electron Diffraction. *Chemical Communications* **2017**, *53* (52), 7018–7021. https://doi.org/10.1039/C7CC03180G.

(19) Plana-Ruiz, S.; Krysiak, Y.; Portillo, J.; Alig, E.; Estradé, S.; Peiró, F.; Kolb, U. Fast-ADT: A Fast and Automated Electron Diffraction Tomography Setup for Structure Determination and Refinement. *Ultramicroscopy* **2020**, *211*, 112951. https://doi.org/10.1016/j.ultramic.2020.112951.

(20) Palatinus, L.; Brázda, P.; Jelínek, M.; Hrdá, J.; Steciuk, G.; Klementová, M. Specifics of the Data Processing of Precession Electron Diffraction Tomography Data and Their Implementation in the Program PETS2.0. *Acta Cryst B* **2019**, *75* (4), 512–522. https://doi.org/10.1107/S2052520619007534.

(21) Stoe & Cie. WinXPow, 2011.

(22) Coelho, A. A. TOPAS and TOPAS-Academic: An Optimization Program Integrating Computer Algebra and Crystallographic Objects Written in C++. *J Appl Cryst* **2018**, *51* (1), 210–218. https://doi.org/10.1107/S1600576718000183.

# 4 Figures and Tables

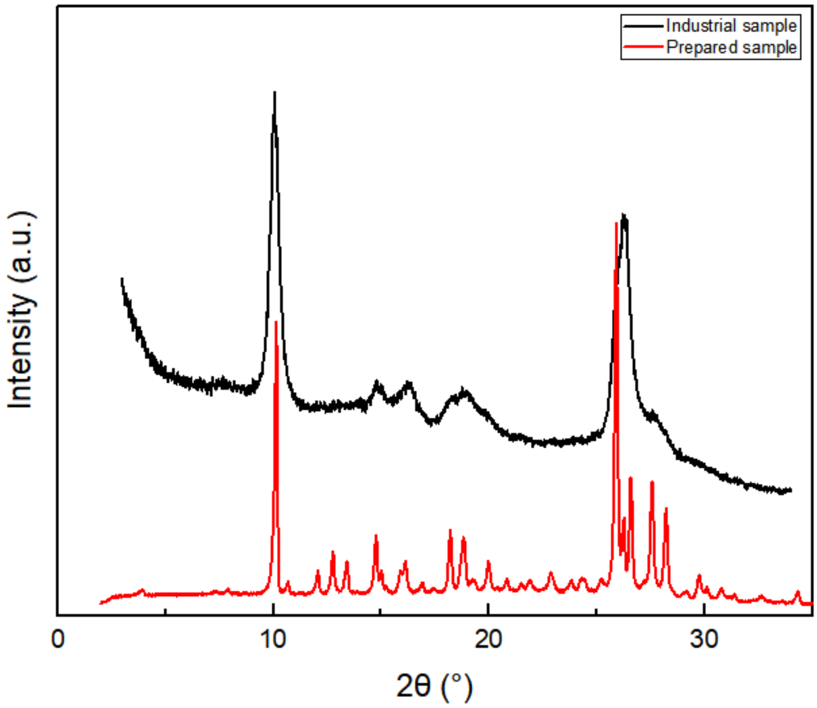


*Figure S3. XRPD pattern of P.O.34 of the industrial (black) and self-prepared (red) sample.*

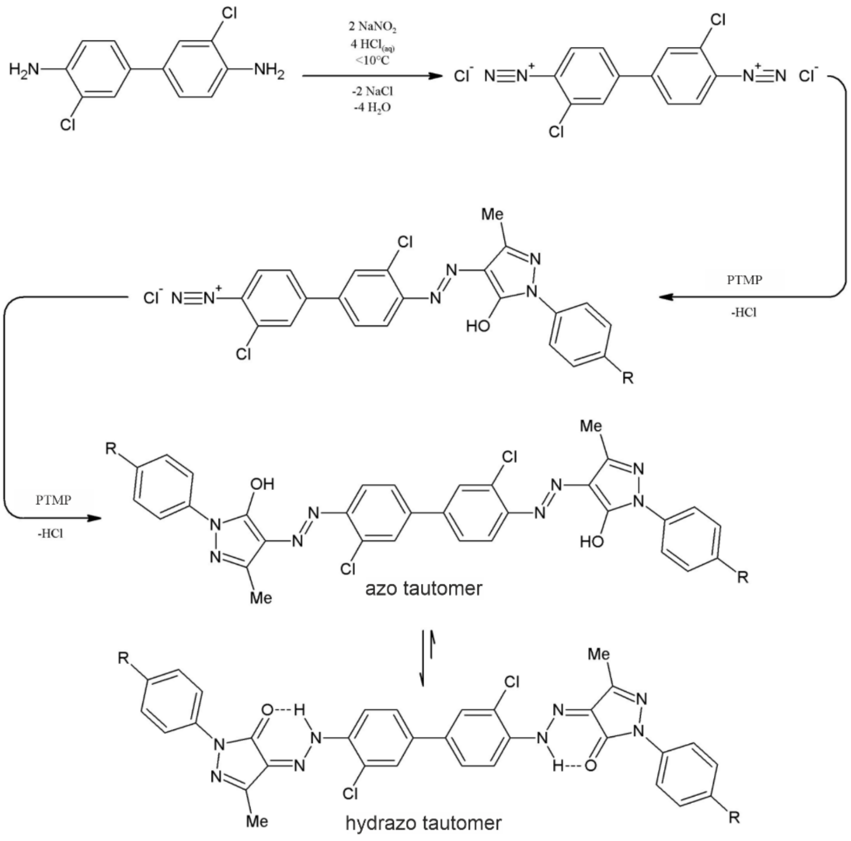


*Figure S4. Reaction scheme of the P.O.34 synthesis. R = $CH_3$*

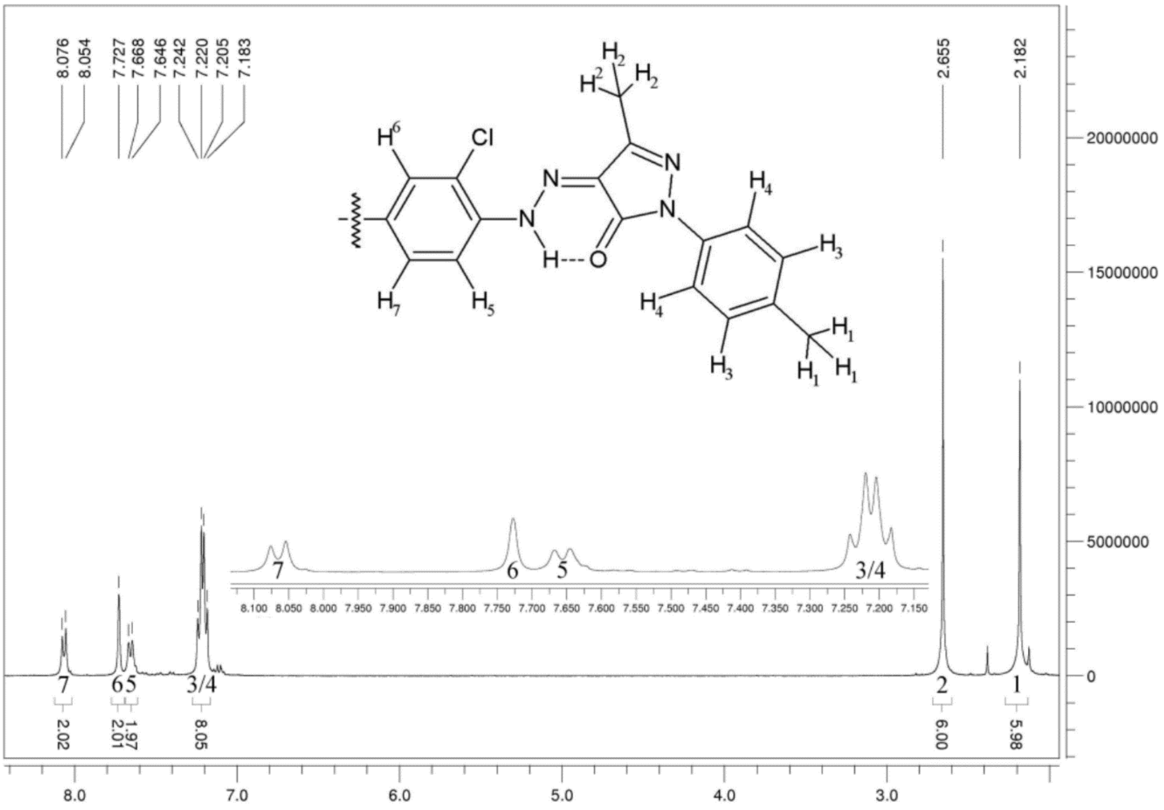

*Figure S5. $^{1}$H-NMR-Spectra (measured in $D_2SO_4$; 10,9 ppm) of P.O.34.*

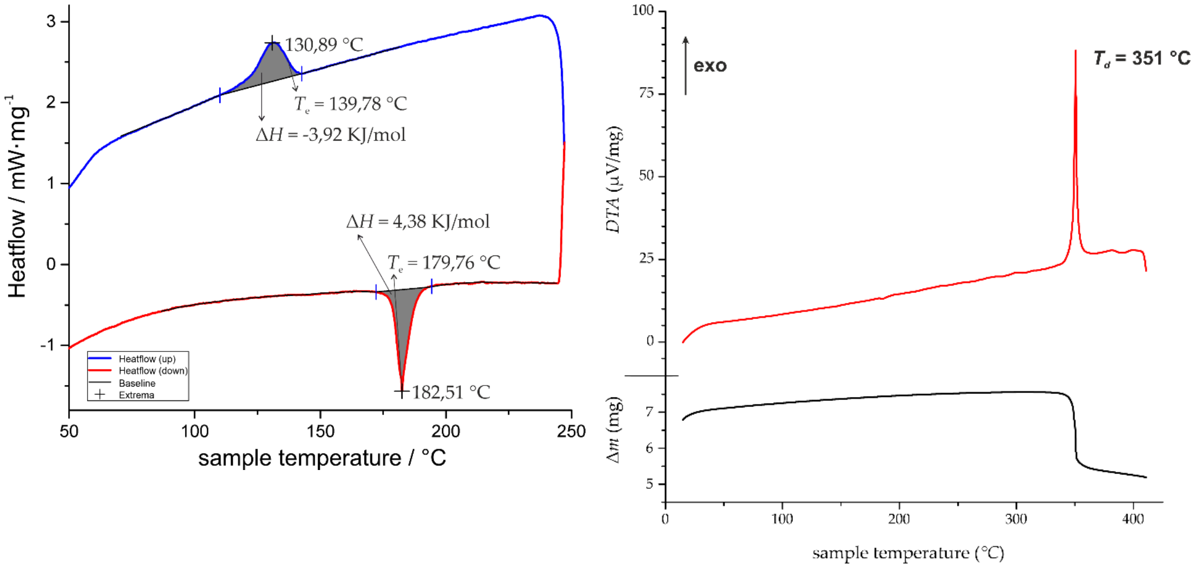


*Figure S6. Differential scanning calorimetry (a) of P.O.34. Red curve shows the progression during the heating and blue curve during the cooling process. (b) Differential thermal and thermogravimetric analysis of P.O.34.*

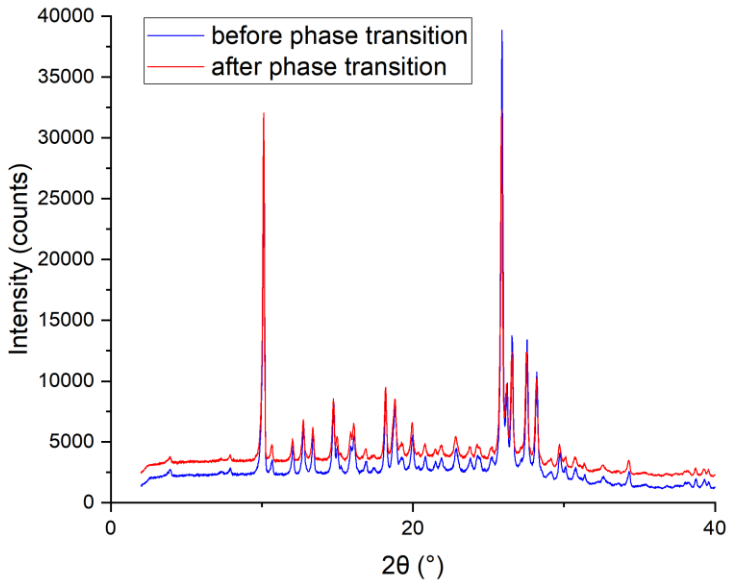


*Figure S7. XRPD pattern of the α-phase of P.O.34 measured at RT before (in blue) and after (in red) heating and cooling the sample from RT to HT (220°C) and down to RT. Red XRPD pattern is shifted by 1000 counts for clarity.*

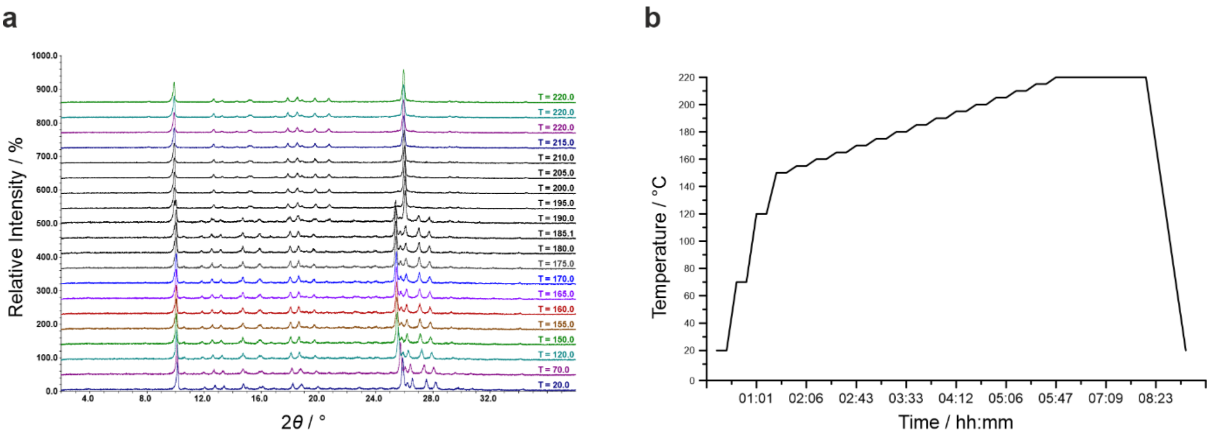


*Figure S8. Powder pattern (a) of the temperature-dependent measurements of P.O.34. (b) Temperature-time curve of the measuring programme.*

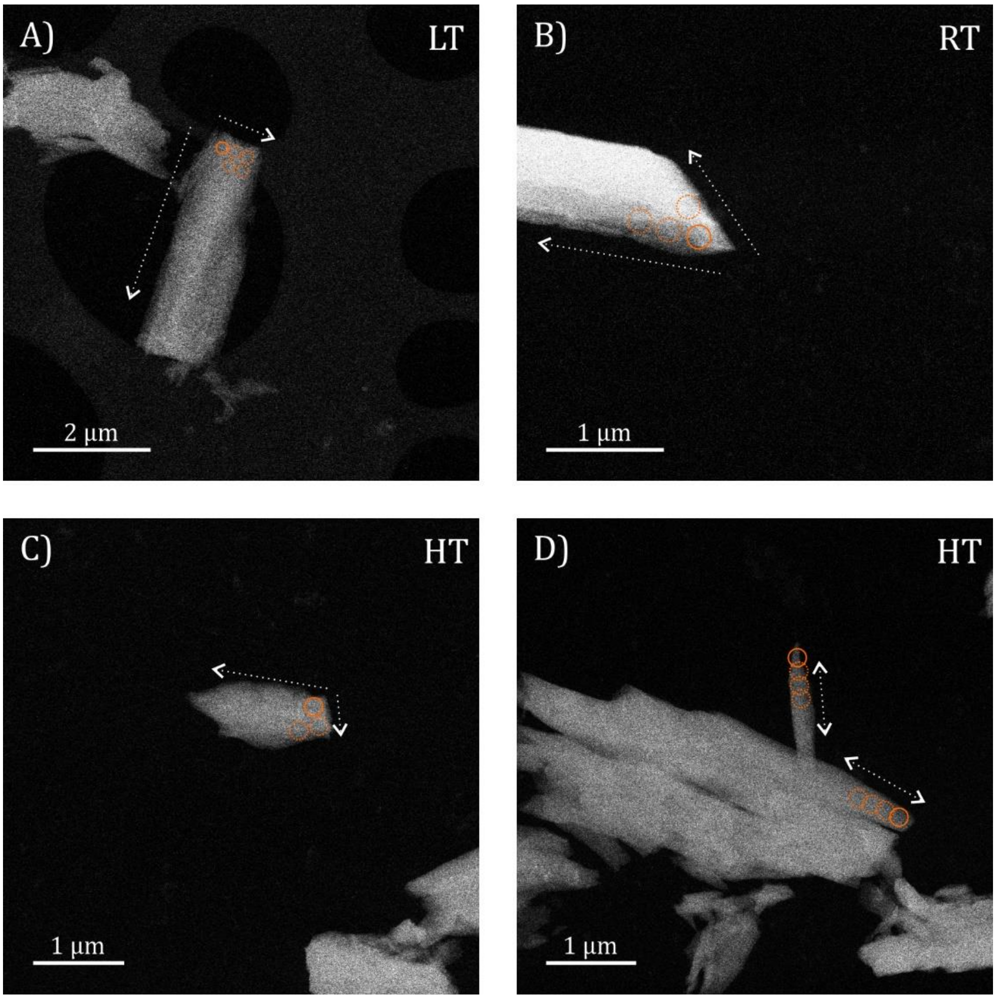


*Figure S9. STEM images of P.O.34 crystals selected for 3D ED measurements at (a) low, (b) room and (c-d) high temperature. The movement of the beam on the particles during the tilt experiment is indicated by the orange circles.*

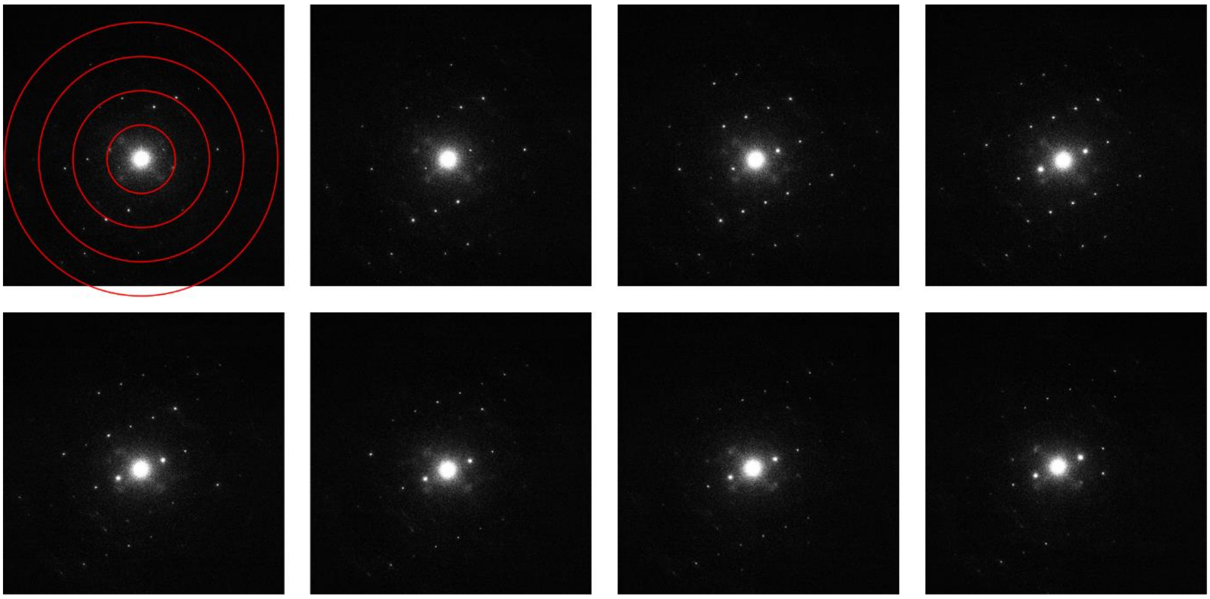

*Figure S10. Exemplary chosen electron diffraction patterns acquired of P.O.34 at 220 °C with Fast-ADT. The red circles indicate resolution shells from 0.25(inner circle) to 1 Å$^{-1}$ (outer circle).*

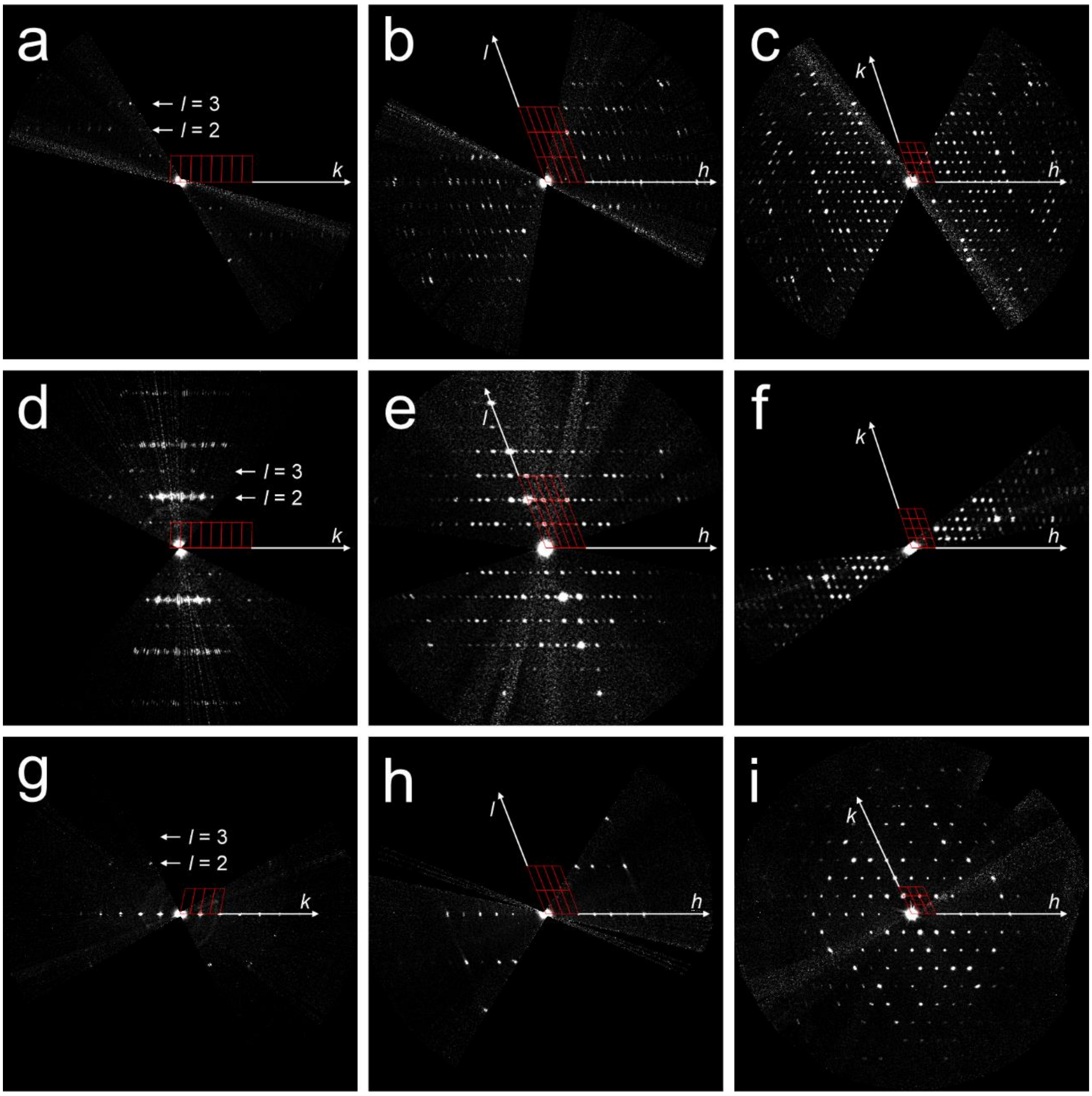


*Figure S11. Observable diffraction space sections 0kl, h0l and hk0 reconstructed from 3D ED data of P.O.34 at (a-c) LT, (d-f) RT and (g-i) HT. The sections of the HT measurement were reconstructed with the F-centred lattice for comparison.*

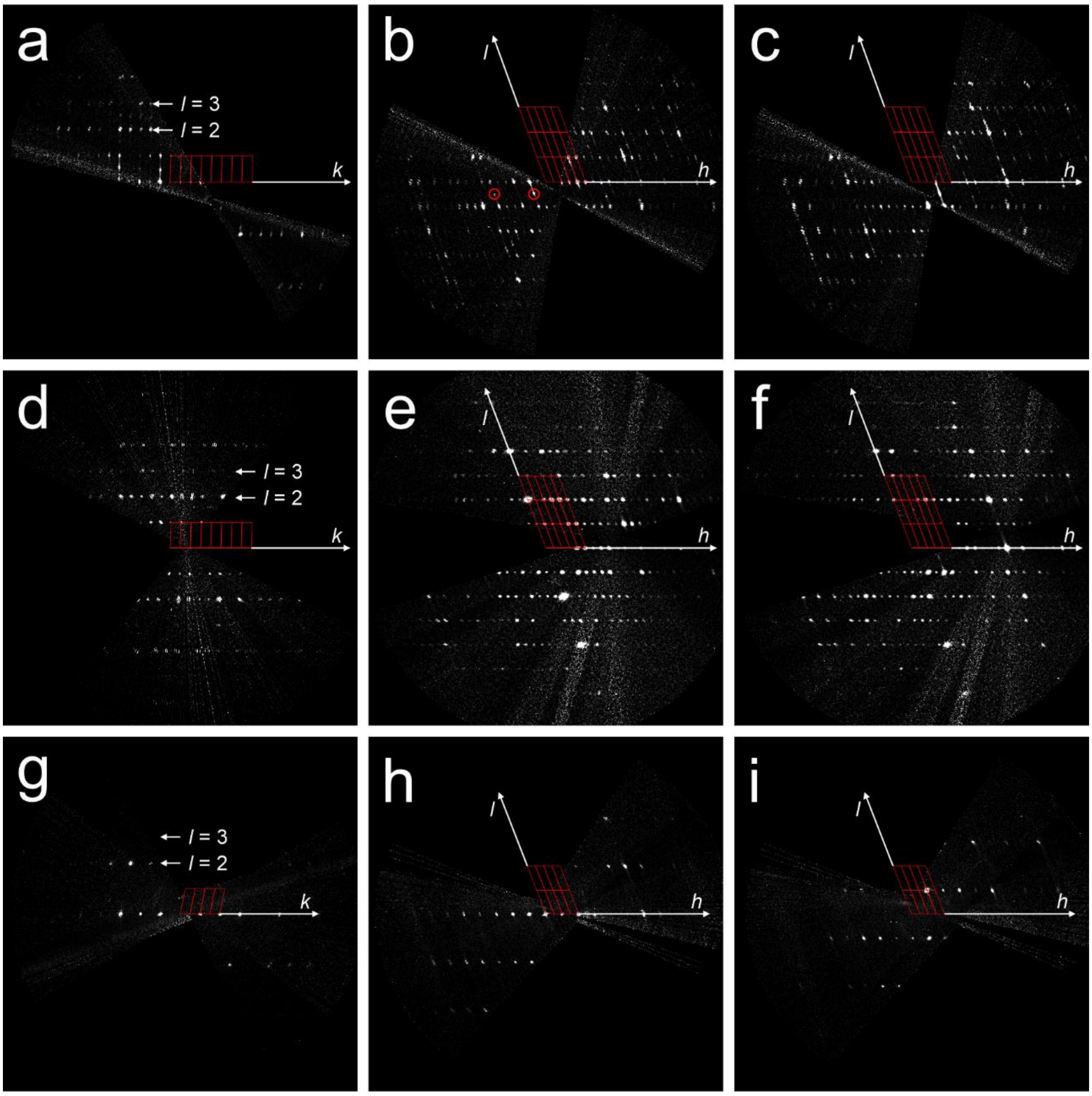


*Figure S12. Observable diffraction space sections 2kl, h2l and h3l reconstructed from 3D ED data of P.O.34 at (a-c) LT, (d-f) RT and (g-i) HT. The sections of the HT measurement were reconstructed with the F-centred lattice for comparison. Strong maxima of diffuse scattering at l = 0.5 of (b) marked with red circles.*

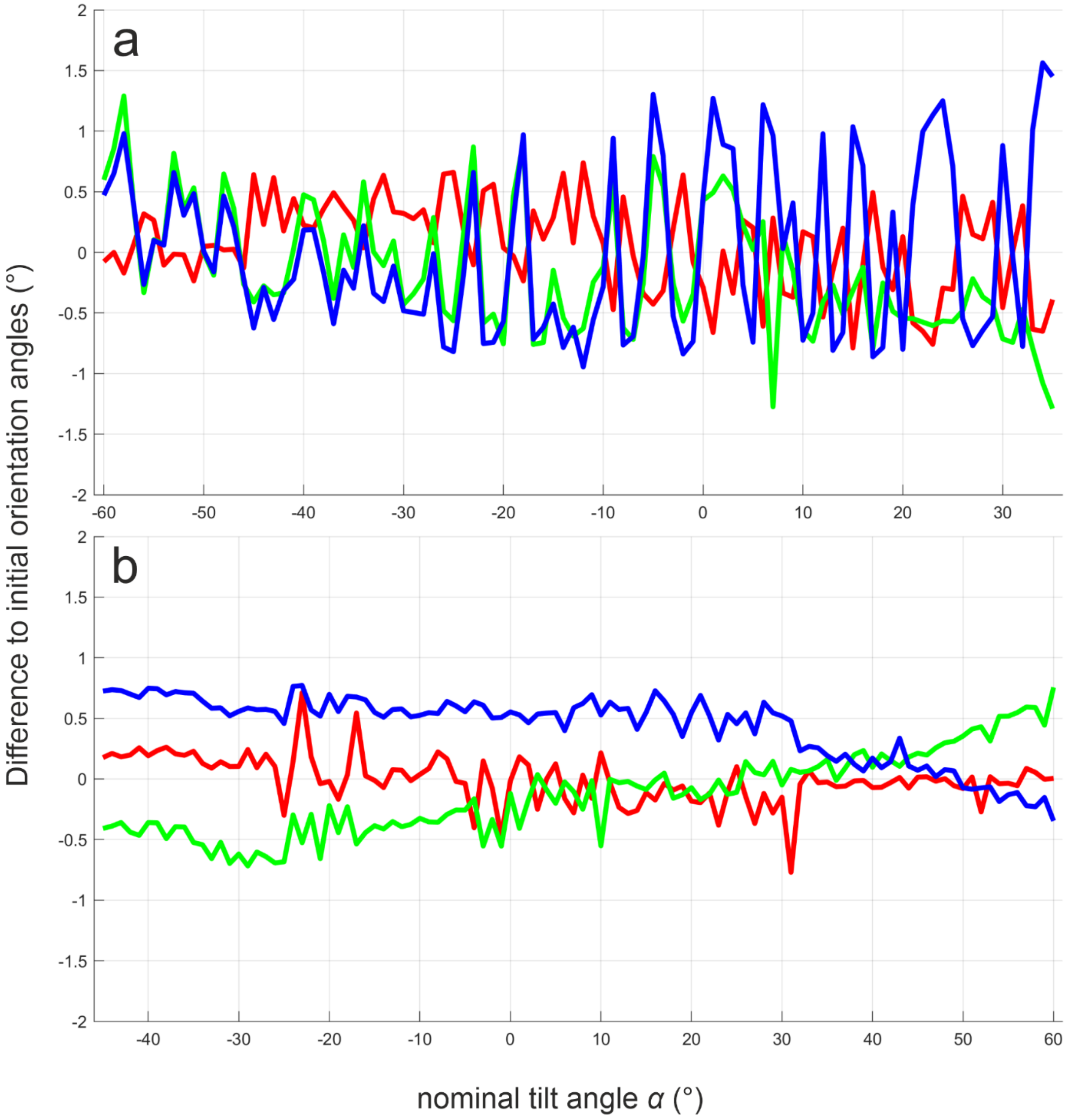


*Figure S13. A plot of the difference to the initial tilt angles α (red), β (green) and γ (blue) obtained by frame orientation refinement of P.O.34 (a) LT and (b) RT 3D ED data.*

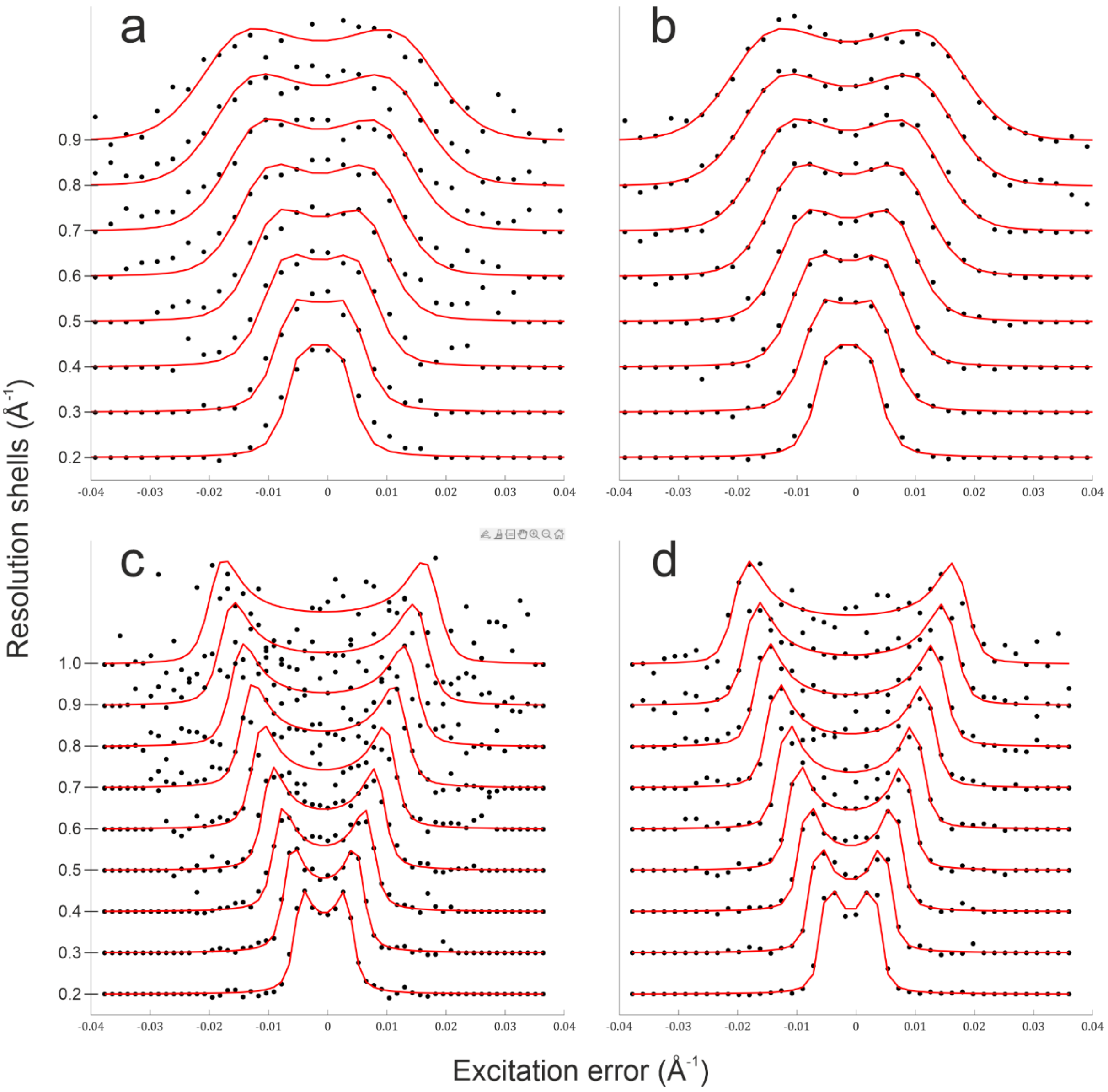


*Figure S14. Averaged rocking-curves of the reflections at different diffraction data resolution intervals (black dots), fitted by the precession-dependent double-peaked profile (red curves). Fit for P.O.34 LT 3D ED data before (a) and after (b) orientation refinement using (a) 0.0015 Å$^{-1}$ and (b) 0.0013 Å$^{-1}$ of reflection width, a mosaicity of 0.3° and precession angle of 1.00° in both cases. Fit for P.O.34 RT 3D ED data before (c) and after (d) orientation refinement using (c) 0.0010 Å$^{-1}$ and (d) 0.0008 Å$^{-1}$ of reflection width, a precession angle of (c) 1.00° and (d) 1.02°, and a mosaicity of 0.07°.*

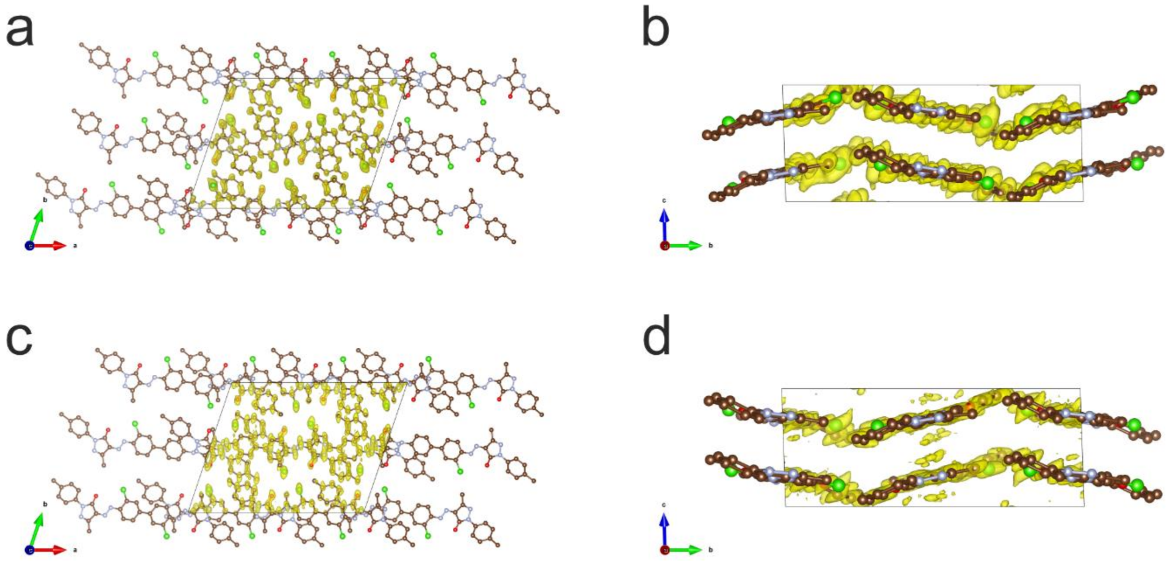


*Figure S15. Results of ab initio structure solutions plotted using VESTA. Potential maps V(**r**) based on structure solution performed with SUPERFLIP for P.O.34 in space group P-1 at (a, b) LT and RT (c, d). Crystal structures plotted with view along (a, c) [001] and (b, d) [100]. Transparent yellow potentials are plotted using 2σ(V(r)) threshold (σ is the standard deviation of map values). Carbon atoms are shown in brown, nitrogen in light blue, oxygen in red and chlorine in green.*

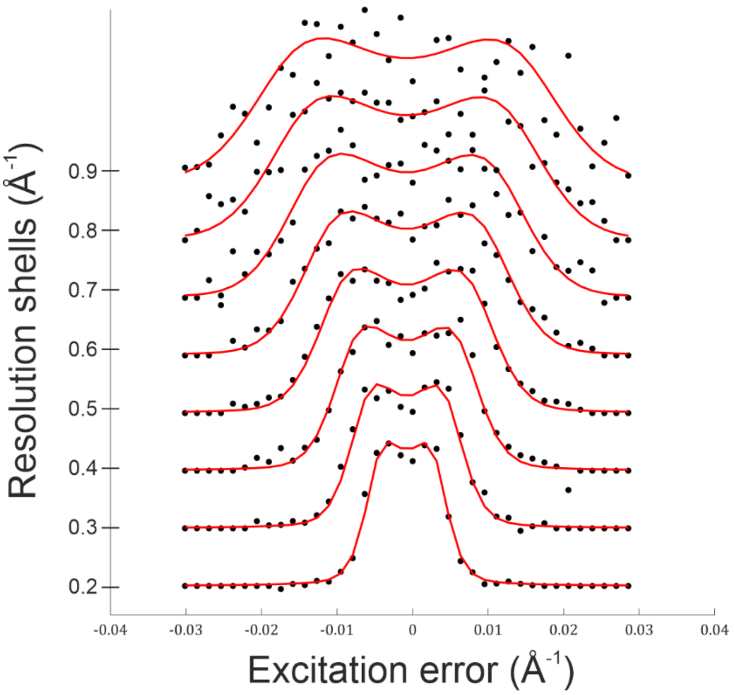


*Figure S16. Averaged rocking-curves of the reflections at different diffraction data resolution intervals (black dots), fitted by the precession-dependent double-peaked profile (red curves). Fit for merged P.O.34 HT 3D ED data using a reflection width of 0.0008 Å$^{-1}$, mosaicity of 0.3° and precession angle of 1.00°.*

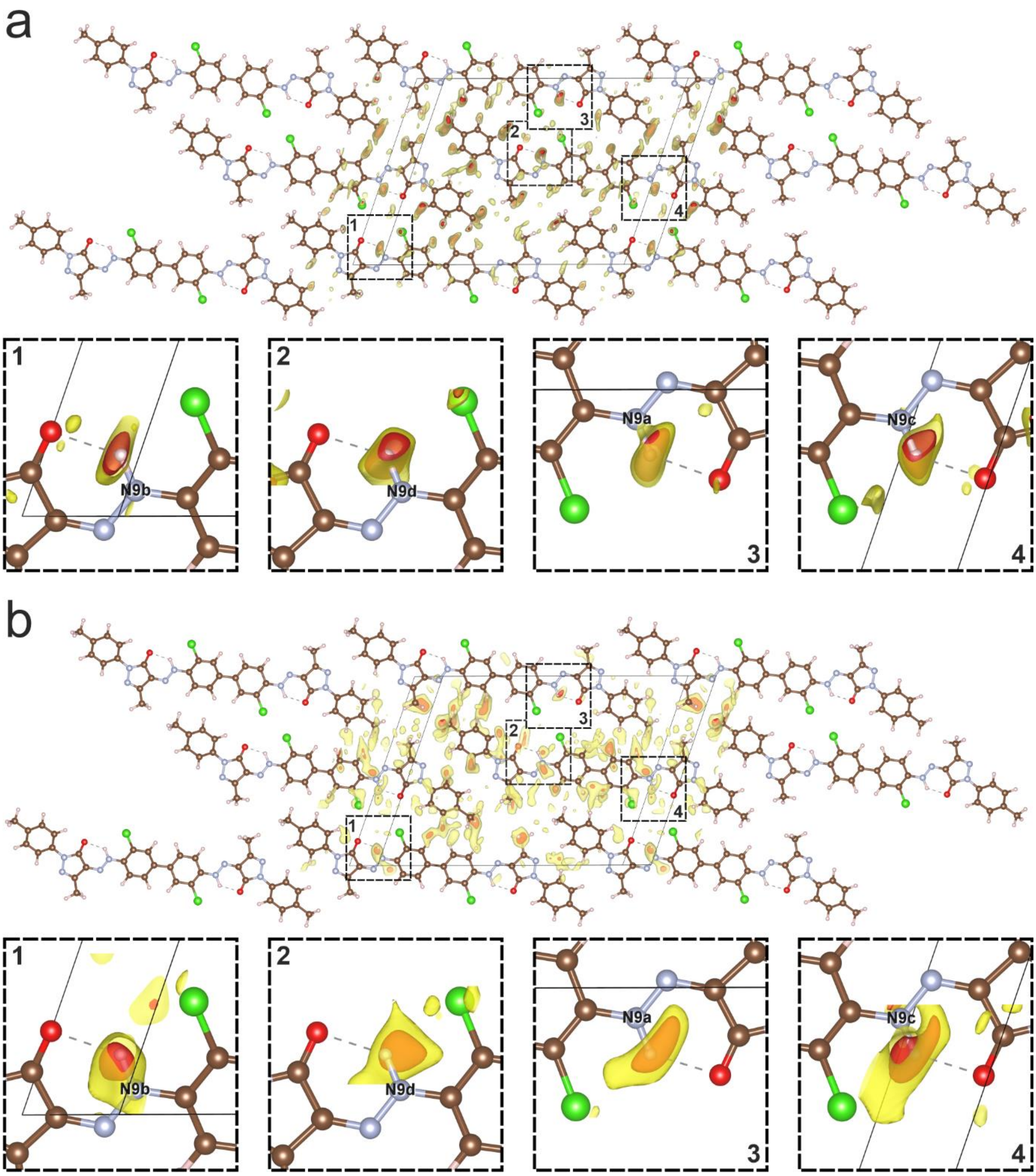


*Figure S17. Superposition of difference Fourier maps and final P.O.34 structure models at (a) LT and (b) RT derived from dynamical refinement. Yellow isosurfaces correspond to the 2σ[ΔV(**r**)] and red isosurfaces to the 3σ[ΔV(**r**)] level. (a,b) View along **c*** of one layer with restricted fractional coordinates in **c**-direction: 0 ≤ z ≤ 0.5 for clarity. The final model was refined with hydrogens. The difference Fourier maps were calculated based on final models without hydrogens. The difference Fourier maps in the enlarged framed areas (1-4) were calculated based on final models without hydrogens at the nitrogen resp. oxygen.*

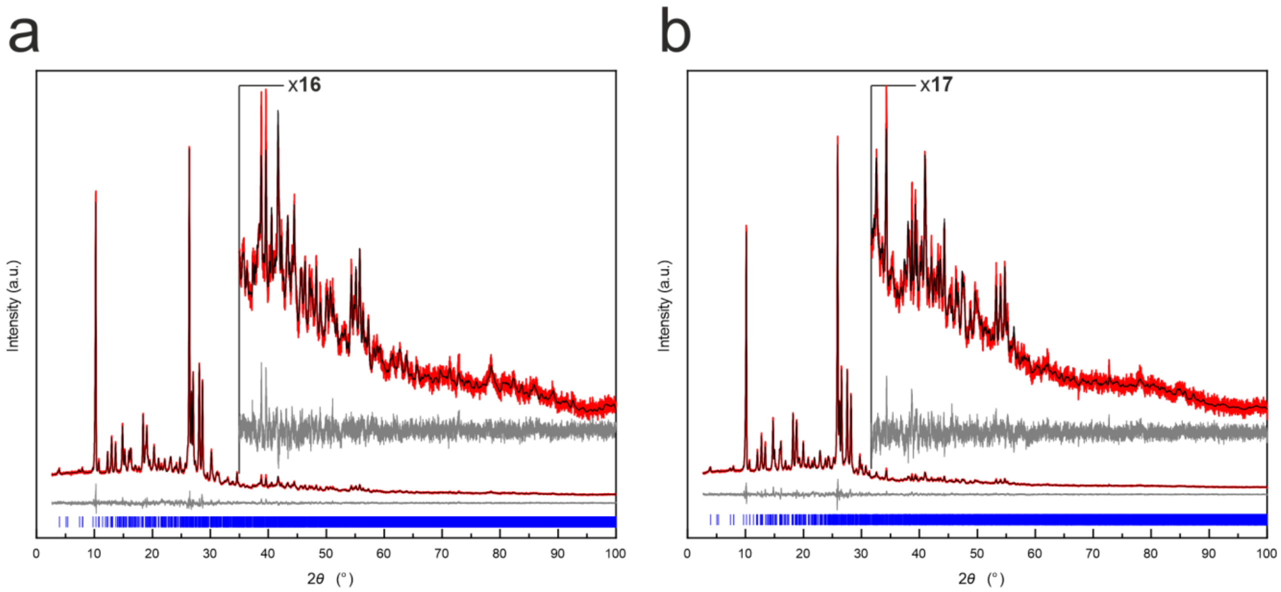


*Figure S18. Rietveld refinement plot (λ = 1.54059 Å) of P.O.34 at (a) low- and (b) room temperature. Red line for measured intensities ($I_O$), black line for fitted profile ($I_C$), and grey line for the difference ($I_O – I_C$).*

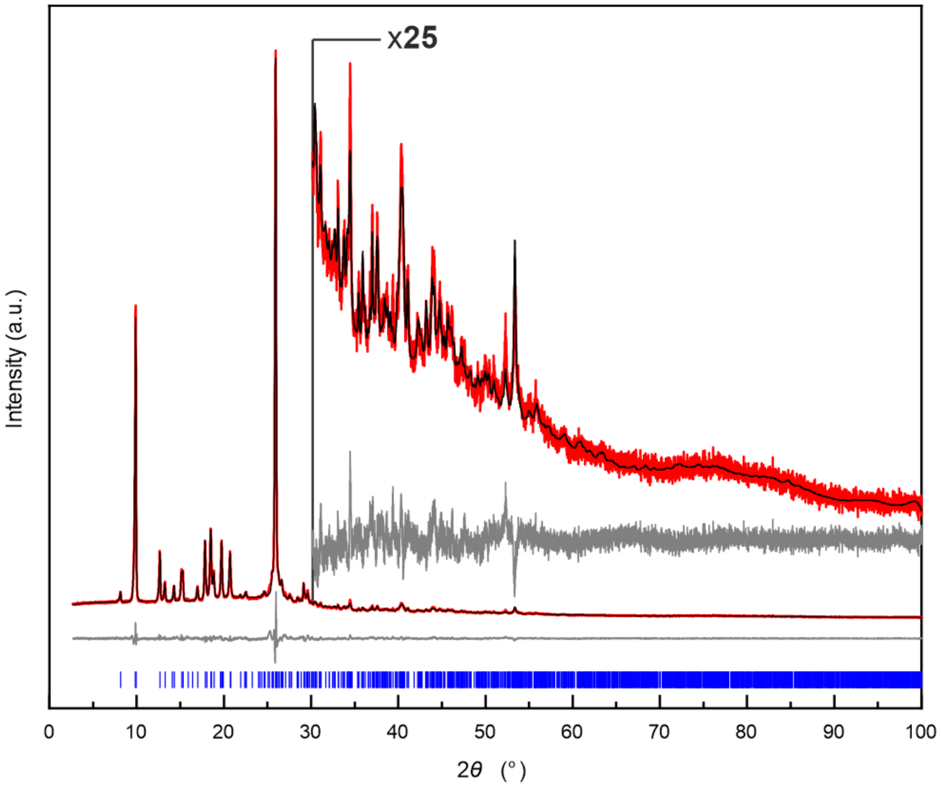


*Figure S19. Rietveld refinement plot (λ = 1.54059 Å) of P.O.34 at 220 °C. Red line for measured intensities ($I_O$), black line for fitted profile ($I_C$), and grey line for the difference ($I_O – I_C$).*

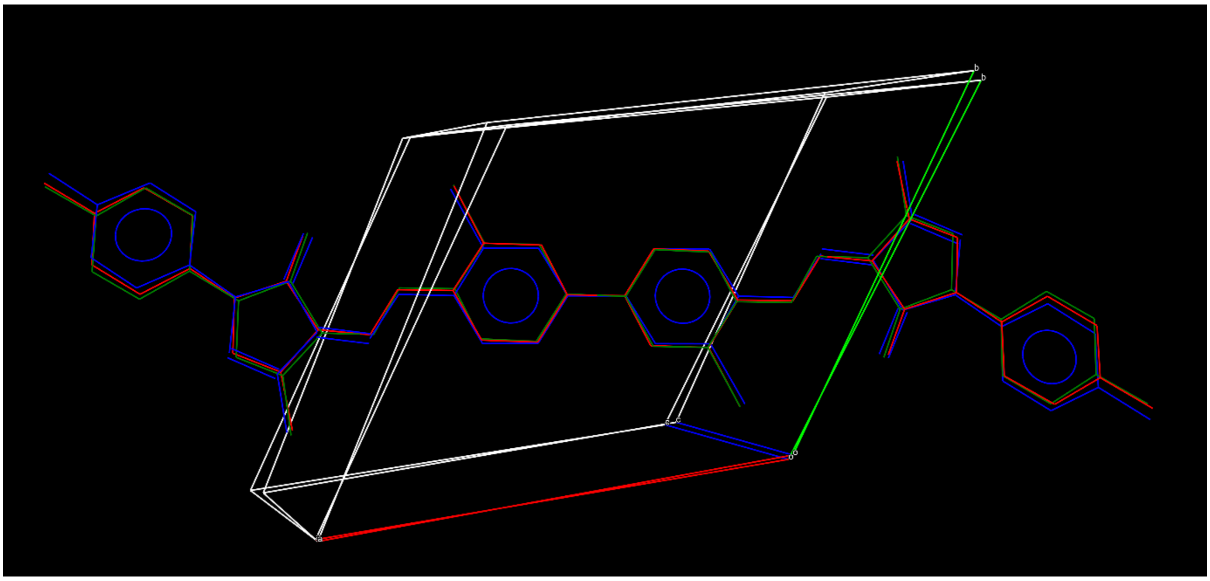

*Figure S20. Superposition of the experimental 3D ED (in red), XRPD (in green) and optimized DFT-D (in blue) crystal structure models of the HT-phase of P.O.34. Hydrogen atoms are omitted for clarity.*

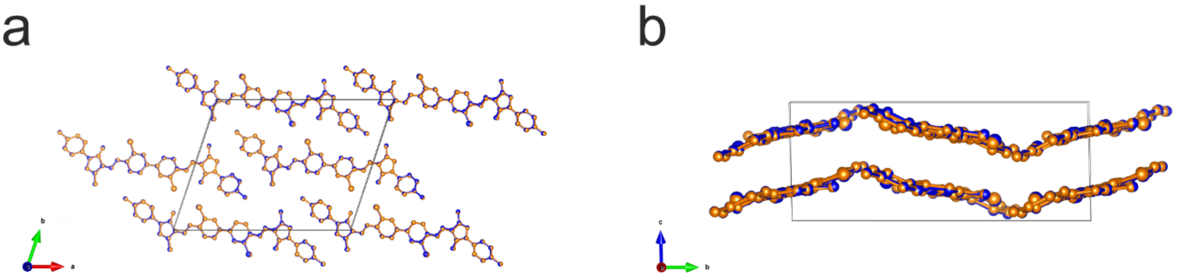


*Figure S21. Superposition of the experimental crystal structure of LT P.O.34 (in orange) on the optimized structure (in blue) calculated by DFT-D. Hydrogen atoms are omitted for clarity. (a) View along [001] with restricted fractional coordinates in* ***c****-direction: 0 ≤ z ≤ 0.5. (b) View along [100].*

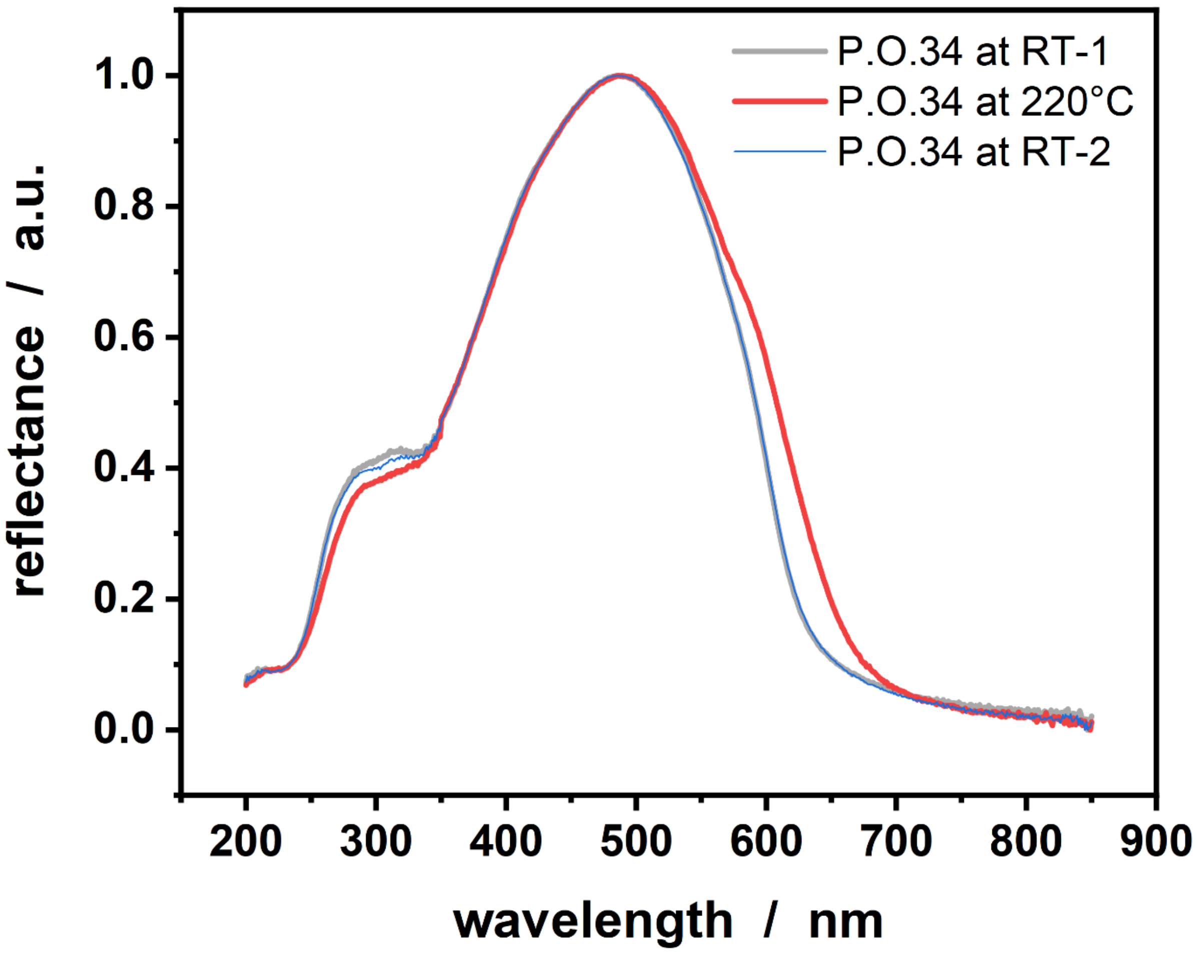


*Figure S22. UV-Vis reflectance spectra measured before heating at room temperature (RT-1 in grey), at 220 °C (in red) and after cooling at room temperature (RT-2 in blue).*

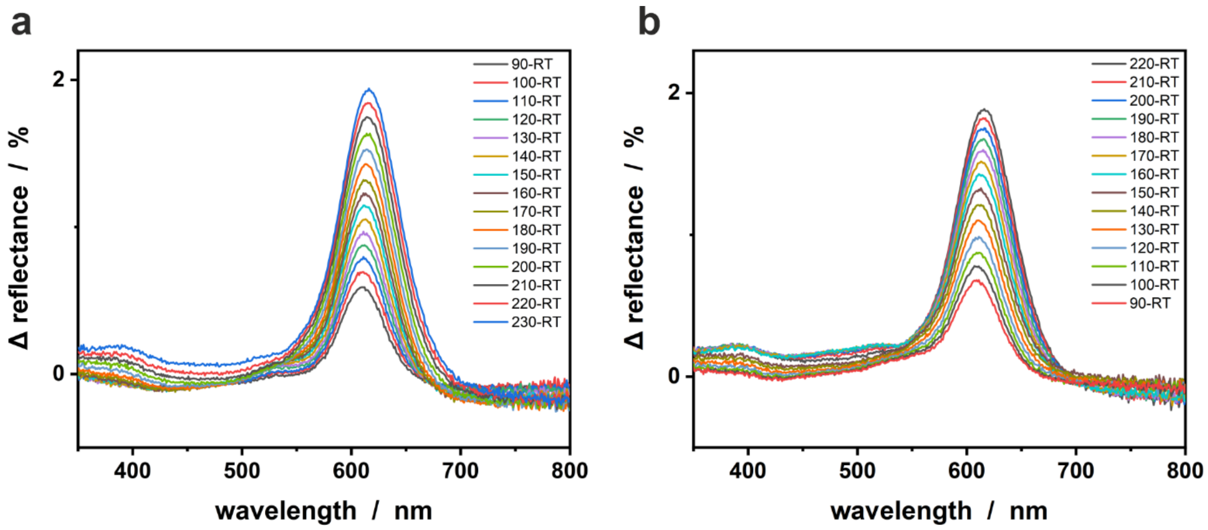


*Figure S23. UV-Vis difference reflectance spectra (referred to RT measurement) shown for (a) heating from 90-230 °C and subsequent (b) cooling from 220-90 °C in 10 °C steps.*

*Table S1. Lattice parameters from 3D ED data of P.O.34 at different temperatures. The primitive cell at 513 K was transformed to a F-centred cell with [-2 0 -1; 0 2 1; 0 0 -1].*

| *T* (K) | Bravais | *a* (Å) | *b* (Å) | *c* (Å) | *α* (°) | *β* (°) | *γ* (°) | *V* (Å³) |
|---|---|---|---|---|---|---|---|---|
| 97 | *P* | 25.09 | 18.24 | 7.20 | 84.7 | 69.0 | 71.2 | 2913 |
| 293 | *P* | 24.97 | 18.46 | 7.42 | 84.8 | 69.1 | 71.2 | 3026 |
| 513 | *F* | 24.93 | 19.54 | 7.43 | 92.9 | 72.2 | 68.0 | 3140 |
| | *P* | 11.87 | 10.28 | 7.43 | 108.3 | 89.5 | 113.0 | 785 |

*Table S2. Relevant parameters of the 3D ED measurement, crystallographic data and refinement results of P.O.34 for LT and RT measurements. The lattice constants listed here were determined on the basis of the electron diffraction data. However, the final data reconstruction and structure determination was performed assuming the lattice constants from the Rietveld refinement, see Table S3.*

| P.O.34 (LT phase) | Low temperature (LT) | Room temperature (RT) |
|---|---|---|
| **Crystal data** | | |
| Crystal system, space group | Triclinic, *P* -1 | |
| *a*, *b*, *c* (Å) | 25.101(9), 18.238(7), 7.232(4) | 25.129(6), 18.45(1), 7.358(2) |
| *α*, *β*, *γ* (°) | 84.62(3), 69.21(3), 70.87(3) | 84.98(3), 69.22(2), 70.89(3) |
| *V* (Å³) | 2923(2) | 3012(2) |
| Empirical formula | $C_{34}H_{28}N_8O_2Cl_2$ | |
| *Z* | 4 | |
| **Data collection** | | |
| Radiation type | electrons, 300 kV | |
| Wavelength (Å) | 0.01970 | |
| Temperature (K) | 97 | 293 |
| Precession angle (deg.) | 1.00 | 1.02 |
| Resolution ($Å^{-1}$) | 1.0 | 1.2 |
| Tilt step (deg.) | 1 | |
| No. of frames | 96 (95)[b] | 106 (103)[b] |
| Completeness (%) | 56 | 61 |
| **Kinematical structure refinement** | | |
| No. of used reflections (obs/all) | 1747/3390 | 1715/3861 |
| $R_1^{obs}/R_1^{all}$, $wR_1^{obs}/wR_1^{all}$ (%) | 15.9/23.4, 15.6/16.2 | 19.4/28.5, 19.5/19.9 |
| goodness of fit (obs/all) | 5.47/3.91 | 7.47/4.84 |
| No. of refined parameters | 301 | 306 |
| $\sigma[\Delta V(\mathbf{r})]$ (e/Å) | 0.166 | 0.209 |
| min[$\Delta V(\mathbf{r})$], max[$\Delta V(\mathbf{r})$] (e/Å) | -0.715, 0.715 | -0.824, 0.824 |
| **Dynamical structure refinement** | | |
| No. of used reflections (obs/all) | 3992/13699 | 4667/18281 |
| Average crystal thickness (nm) | 51 | 89 |
| $g_{max}$, $Sg_{max}M$, $RSg_{max}$, $DSg_{min}$, N | 1.1, 0.01, 0.6, 1.7e-3, 120 | 1.1, 0.01, 0.8, 1.7e-3, 120 |
| $R_1^{obs}/R_1^{all}$, $wR_1^{obs}/wR_1^{all}$ (%) | 10.5/22.6, 10.5/12.1 | 10.0/25.5, 10.3/11.9 |
| goodness of fit (obs/all) | 2.51/1.53 | 2.55/1.48 |
| No. of refined parameters[a] | 300+95 | 323+103 |
| $\sigma[\Delta V(\mathbf{r})]$ (e/Å) | 0.090 | 0.096 |
| min[$\Delta V(\mathbf{r})$], max[$\Delta V(\mathbf{r})$] (e/Å) | -0.399, 0.399 | -0.366, 0.415 |

[a] *Split into the terms of the sum structural parameters and scale factors.*
[b] *Number of frames used for structure refinement in brackets.*

*Table S3. Relevant parameters of the 3D ED measurement, crystallographic data and refinement results of P.O.34 for high-temperature measurements. The primitive cell at 513 K was transformed to a F-centred cell with [-2 0 -1; 0 2 1; 0 0 -1].*

| P.O.34 (HT phase) | High temperature (HT) | |
|---|---|---|
| **Crystal data** | | |
| Crystal system, space group | Triclinic, *P* -1 | Triclinic, *F* -1 |
| *a*, *b*, *c* (Å) | 11.915(4), 10.348(4), 7.394(4) | 25.16(1), 19.666(7), 7.400(7) |
| *α*, *β*, *γ* (°) | 108.34(4), 88.30(3), 114.21(3) | 92.60(5), 71.26(5), 67.35(3) |
| *V* (Å³) | 784.1(7) | 3140(4) |
| Empirical formula | $C_{34}H_{28}N_8O_2Cl_2$ | |
| *Z* | 1 | 4 |
| **Data collection** | | |
| Radiation type | electrons, 300 kV | |
| Wavelength (Å) | 0.01970 | |
| Temperature (K) | 493 | |
| Precession angle (deg.) | 1.00 | |
| Resolution (Å$^{-1}$) | 1.0 | |
| Tilt step (°) | 1 | |
| $\alpha_{min}$, $\alpha_{max}$ (°) #1,2 and 3 | -40, 35 | |
| No. of used frames | 222 | |
| Completeness (%) | 72 | |
| **Kinematical structure refinement** | | |
| No. of used reflections (obs/all) | 368/1147 | |
| $R_1^{obs}/R_1^{all}$, $wR_1^{obs}/wR_1^{all}$ (%) | 12.8/26.6, 12.1/26.6 | |
| goodness of fit (obs/all) | 5.67/3.06 | |
| No. of refined parameters | 80 | |
| $\sigma[\Delta V(\mathbf{r})]$ (e/Å) | 0.082 | |
| min[$\Delta V(\mathbf{r})$], max[$\Delta V(\mathbf{r})$] (e/Å) | -0.360, 0.312 | |

*Table S4. Results and crystallographic data of the Rietveld refinements of P.O.34 at -180°C (LT), room-temperature (RT) and 220°C (HT).*

| P.O.34 | LT | RT | HT |
|---|---|---|---|
| Crystal system, space group | Triclinic, *P*-1 | | |
| *a* (Å) | 25.108(2) | 25.1377(17) | 11.9124(6) |
| *b* (Å) | 18.2350(14) | 18.4324(16) | 10.3496(6) |
| *c* (Å) | 7.2177(4) | 7.3554(4) | 7.3858(4) |
| *α* (°) | 84.760(3) | 85.004(3) | 108.348(3) |
| *β* (°) | 69.218(6) | 69.185(6) | 88.216(3) |
| *γ* (°) | 70.867(5) | 70.826(5) | 114.226(3) |
| *V* (Å³) | 2917.7(4) | 3007.2(4) | 783.11(8) |
| Empirical formula | $C_{34}H_{28}N_8O_2Cl_2$ | | |
| *Z, Z'* | 4, 2 | 4, 2 | 1, 1/2 |
| $B_{iso}$-C,N,O (Å$^2$) | 0.43 | 2.31 | 6.84 |
| $B_{iso}$-Cl (Å$^2$) | 1.5 * $B_{iso}$-C,N,O | | 1.3 * $B_{iso}$-C,N,O |
| $B_{iso}$-H (Å$^2$) | 1.2 * $B_{iso}$-C,N,O | | |
| $\theta_{max}$ (°) | 50 | | |
| $R_p$ (%) | 2.81 | 2.91 | 3.22 |

| | | | |
|---|---|---|---|
| $R_{wp}$ (%) | 3.62 | 3.74 | 4.20 |
| $R_{exp}$ (%) | 2.40 | 2.44 | 2.43 |
| *Gof* | 1.50 | 1.54 | 1.73 |

# 5 Crystal structure data of P.O.34 determined by Rietveld refinement based on PXRD data recorded at 93, 293 and 493 K.

```
#======================================================================
# CRYSTAL DATA
#----------------------------------------------------------------------
data_LT_phase_93K

_chemical_name_common                  'Pigment Orange 34 - LT phase at 93 K'
_cell_length_a                         25.108(2)
_cell_length_b                         18.2350(14)
_cell_length_c                         7.2177(4)
_cell_angle_alpha                      84.760(3)
_cell_angle_beta                       69.218(6)
_cell_angle_gamma                      70.867(5)
_cell_volume                           2917.744346
_space_group_name_H-M_alt              'P -1'
_space_group_IT_number                 2

loop_
_space_group_symop_operation_xyz
   'x, y, z'
   '-x, -y, -z'

loop_
   _atom_site_label
   _atom_site_occupancy
   _atom_site_fract_x
   _atom_site_fract_y
   _atom_site_fract_z
   _atom_site_adp_type
   _atom_site_U_iso_or_equiv
   _atom_site_type_symbol
   C1a      1.0    0.570(12)    0.006(13)    0.23(3)     Uiso  0.005480 C
   C2a      1.0    0.617(11)   -0.072(13)    0.20(3)     Uiso  0.005480 C
   C3a      1.0    0.598(10)    0.061(13)    0.25(2)     Uiso  0.005480 C
   N4a      1.0    0.511(9)     0.021(11)    0.24(2)     Uiso  0.005480 N
   N5a      1.0    0.667(8)    -0.056(11)    0.20(2)     Uiso  0.005480 N
   O6a      1.0    0.615(6)    -0.136(8)     0.176(16)   Uiso  0.005480 O
   N7a      1.0    0.654(8)     0.025(10)    0.24(2)     Uiso  0.005480 N
   C8a      1.0    0.571(11)    0.149(14)    0.29(3)     Uiso  0.005480 C
   N9a      1.0    0.499(8)    -0.039(11)    0.22(3)     Uiso  0.005480 N
   C10a     1.0    0.726(9)    -0.109(13)    0.17(3)     Uiso  0.005480 C
   H11a     1.0    0.60(7)      0.17(9)      0.2(2)      Uiso  0.006580 H
   H12a     1.0    0.54(7)      0.17(9)      0.2(2)      Uiso  0.006580 H
   H13a     1.0    0.56(7)      0.16(8)      0.43(19)    Uiso  0.006580 H
   C14a     1.0    0.440(10)   -0.028(14)    0.23(3)     Uiso  0.005480 C
   H15a     1.0    0.53(8)     -0.09(8)      0.2(2)      Uiso  0.006580 H
   C16a     1.0    0.747(12)   -0.182(13)    0.07(3)     Uiso  0.005480 C
   C17a     1.0    0.763(9)    -0.083(12)    0.24(3)     Uiso  0.005480 C
   C18a     1.0    0.399(12)    0.047(13)    0.25(3)     Uiso  0.005480 C
   C19a     1.0    0.422(10)   -0.091(11)    0.21(3)     Uiso  0.005480 C
   C20a     1.0    0.805(10)   -0.230(13)    0.04(3)     Uiso  0.005480 C
   H21a     1.0    0.72(7)     -0.20(8)      0.0(2)      Uiso  0.006580 H
   C22a     1.0    0.821(12)   -0.131(13)    0.21(3)     Uiso  0.005480 C
   H23a     1.0    0.75(7)     -0.03(9)      0.31(18)    Uiso  0.006580 H
   C24a     1.0    0.340(11)    0.059(12)    0.25(3)     Uiso  0.005480 C
   H25a     1.0    0.41(8)      0.09(9)      0.3(2)      Uiso  0.006580 H
   C26a     1.0    0.363(11)   -0.079(13)    0.21(3)     Uiso  0.005480 C
   Cl27a    1.0    0.472(3)    -0.186(3)     0.178(8)    Uiso  0.008230 Cl
   C28a     1.0    0.841(10)   -0.204(13)    0.11(3)     Uiso  0.005480 C
   H29a     1.0    0.82(7)     -0.28(8)     -0.02(19)    Uiso  0.006580 H
   H30a     1.0    0.85(7)     -0.12(9)      0.3(2)      Uiso  0.006580 H
   C31a     1.0    0.322(11)   -0.004(13)    0.24(3)     Uiso  0.005480 C
   H32a     1.0    0.31(6)      0.11(9)      0.3(2)      Uiso  0.006580 H
   H33a     1.0    0.35(7)     -0.12(8)      0.2(2)      Uiso  0.006580 H
   C34a     1.0    0.903(11)   -0.255(12)    0.08(3)     Uiso  0.005480 C
   H36a     1.0    0.91(7)     -0.30(8)      0.00(19)    Uiso  0.006580 H
   H37a     1.0    0.93(6)     -0.23(8)      0.01(19)    Uiso  0.006580 H
   H38a     1.0    0.91(7)     -0.27(9)      0.21(18)    Uiso  0.006580 H
   C1b      1.0    0.012(10)   -0.005(12)    0.24(3)     Uiso  0.005480 C
   C2b      1.0   -0.036(10)    0.070(14)    0.28(3)     Uiso  0.005480 C
   C3b      1.0   -0.014(10)   -0.060(11)    0.21(3)     Uiso  0.005480 C
   N4b      1.0    0.071(8)    -0.017(9)     0.23(2)     Uiso  0.005480 N
   N5b      1.0   -0.084(8)     0.055(10)    0.27(2)     Uiso  0.005480 N
   O6b      1.0   -0.034(6)     0.132(8)     0.317(17)   Uiso  0.005480 O
   N7b      1.0   -0.069(8)    -0.026(9)     0.23(2)     Uiso  0.005480 N
   C8b      1.0    0.017(12)   -0.146(15)    0.16(3)     Uiso  0.005480 C
   N9b      1.0    0.081(9)     0.043(10)    0.26(3)     Uiso  0.005480 N
   C10b     1.0   -0.145(10)    0.105(12)    0.31(3)     Uiso  0.005480 C
   H11b     1.0   -0.01(7)     -0.17(9)      0.2(2)      Uiso  0.006580 H
   H12b     1.0    0.05(7)     -0.15(9)      0.02(19)    Uiso  0.006580 H
   H13b     1.0    0.04(8)     -0.17(9)      0.25(18)    Uiso  0.006580 H
   C14b     1.0    0.141(10)    0.032(13)    0.25(3)     Uiso  0.005480 C
   H15b     1.0    0.05(7)      0.09(9)      0.3(2)      Uiso  0.006580 H
   C16b     1.0   -0.182(11)    0.078(13)    0.24(3)     Uiso  0.005480 C
   C17b     1.0   -0.169(11)    0.177(13)    0.42(3)     Uiso  0.005480 C
   C18b     1.0    0.186(11)   -0.039(14)    0.19(3)     Uiso  0.005480 C
   C19b     1.0    0.156(10)    0.092(12)    0.31(3)     Uiso  0.005480 C
   C20b     1.0   -0.241(9)     0.121(12)    0.28(3)     Uiso  0.005480 C
   H21b     1.0   -0.17(7)      0.03(8)      0.17(18)    Uiso  0.006580 H
   C22b     1.0   -0.229(9)     0.220(10)    0.45(3)     Uiso  0.005480 C
   H23b     1.0   -0.14(7)      0.20(8)      0.47(19)    Uiso  0.006580 H
   C24b     1.0    0.245(12)   -0.051(12)    0.18(3)     Uiso  0.005480 C
   H25b     1.0    0.18(8)     -0.08(10)     0.1(2)      Uiso  0.006580 H
   C26b     1.0    0.215(11)    0.080(13)    0.31(3)     Uiso  0.005480 C
   Cl27b    1.0    0.102(3)     0.182(3)     0.396(6)    Uiso  0.008230 Cl
   C28b     1.0   -0.265(10)    0.193(12)    0.39(3)     Uiso  0.005480 C
   H29b     1.0   -0.27(7)      0.10(11)     0.2(2)      Uiso  0.006580 H
   H30b     1.0   -0.25(7)      0.27(9)      0.53(17)    Uiso  0.006580 H
   C31b     1.0    0.259(12)    0.009(14)    0.24(3)     Uiso  0.005480 C
   H32b     1.0    0.28(8)     -0.10(9)      0.1(2)      Uiso  0.006580 H
   H33b     1.0    0.22(7)      0.12(9)      0.3(2)      Uiso  0.006580 H
   C34b     1.0   -0.331(12)    0.241(13)    0.43(3)     Uiso  0.005480 C
   H36b     1.0   -0.34(8)      0.23(9)      0.32(17)    Uiso  0.006580 H
   H37b     1.0   -0.36(7)      0.23(8)      0.55(17)    Uiso  0.006580 H
   H38b     1.0   -0.33(7)      0.30(9)      0.4(2)      Uiso  0.006580 H
   C1c      1.0    1.033(11)    0.509(13)    0.26(3)     Uiso  0.005480 C
   C2c      1.0    1.074(12)    0.435(13)    0.29(3)     Uiso  0.005480 C
   C3c      1.0    1.063(11)    0.564(12)    0.24(3)     Uiso  0.005480 C
   N4c      1.0    0.977(8)     0.523(10)    0.25(2)     Uiso  0.005480 N
   N5c      1.0    1.124(8)     0.452(10)    0.28(2)     Uiso  0.005480 N
   O6c      1.0    1.069(6)     0.370(8)     0.308(19)   Uiso  0.005480 O
   N7c      1.0    1.116(8)     0.532(9)     0.26(2)     Uiso  0.005480 N
   C8c      1.0    1.038(11)    0.650(14)    0.21(3)     Uiso  0.005480 C
   N9c      1.0    0.962(9)     0.462(11)    0.26(2)     Uiso  0.005480 N
   C10c     1.0    1.176(11)    0.400(13)    0.31(3)     Uiso  0.005480 C
   H11c     1.0    1.05(8)      0.68(10)     0.3(2)      Uiso  0.006580 H
   H12c     1.0    1.05(8)      0.66(8)      0.1(2)      Uiso  0.006580 H
   H13c     1.0    0.99(7)      0.67(10)     0.3(2)      Uiso  0.006580 H
   C14c     1.0    0.904(10)    0.471(12)    0.26(3)     Uiso  0.005480 C
   H15c     1.0    0.99(8)      0.42(9)      0.3(2)      Uiso  0.006580 H
   C16c     1.0    1.228(11)    0.422(12)    0.24(3)     Uiso  0.005480 C
   C17c     1.0    1.175(10)    0.329(14)    0.40(3)     Uiso  0.005480 C
   C18c     1.0    0.869(12)    0.545(14)    0.22(3)     Uiso  0.005480 C
   C19c     1.0    0.881(10)    0.408(12)    0.30(3)     Uiso  0.005480 C
   C20c     1.0    1.280(11)    0.373(14)    0.27(3)     Uiso  0.005480 C
   H21c     1.0    1.23(6)      0.47(9)      0.2(2)      Uiso  0.006580 H
   C22c     1.0    1.228(10)    0.280(13)    0.43(3)     Uiso  0.005480 C
   H23c     1.0    1.14(7)      0.32(9)      0.4(2)      Uiso  0.006580 H
   C24c     1.0    0.812(12)    0.554(12)    0.22(3)     Uiso  0.005480 C
   H25c     1.0    0.88(7)      0.59(9)      0.2(2)      Uiso  0.006580 H
   C26c     1.0    0.824(12)    0.418(13)    0.30(3)     Uiso  0.005480 C
   Cl27c    1.0    0.923(2)     0.317(3)     0.351(7)    Uiso  0.008230 Cl
   C28c     1.0    1.280(10)    0.302(12)    0.36(2)     Uiso  0.005480 C
   H29c     1.0    1.32(7)      0.39(10)     0.22(18)    Uiso  0.006580 H
   H30c     1.0    1.23(7)      0.23(9)      0.49(19)    Uiso  0.006580 H
   C31c     1.0    0.789(11)    0.491(17)    0.26(3)     Uiso  0.005480 C
   H32c     1.0    0.79(8)      0.60(9)      0.2(2)      Uiso  0.006580 H
   H33c     1.0    0.81(7)      0.38(9)      0.3(2)      Uiso  0.006580 H
   C34c     1.0    1.337(11)    0.249(15)    0.39(3)     Uiso  0.005480 C
   H36c     1.0    1.37(7)      0.27(10)     0.3(2)      Uiso  0.006580 H
   H37c     1.0    1.33(7)      0.24(9)      0.53(17)    Uiso  0.006580 H
   H38c     1.0    1.35(8)      0.20(8)      0.3(2)      Uiso  0.006580 H
   C1d      1.0    0.480(10)    0.493(13)    0.24(3)     Uiso  0.005480 C
   C2d      1.0    0.438(10)    0.567(13)    0.21(3)     Uiso  0.005480 C
   C3d      1.0    0.450(12)    0.437(13)    0.25(3)     Uiso  0.005480 C
   N4d      1.0    0.535(8)     0.480(9)     0.26(2)     Uiso  0.005480 N
   N5d      1.0    0.389(8)     0.548(11)    0.21(2)     Uiso  0.005480 N
   O6d      1.0    0.446(7)     0.632(7)     0.197(17)   Uiso  0.005480 O
   N7d      1.0    0.398(8)     0.468(9)     0.23(2)     Uiso  0.005480 N
   C8d      1.0    0.474(10)    0.352(13)    0.28(3)     Uiso  0.005480 C
   N9d      1.0    0.552(8)     0.539(10)    0.25(3)     Uiso  0.005480 N
   C10d     1.0    0.336(10)    0.598(12)    0.18(3)     Uiso  0.005480 C
   H11d     1.0    0.45(8)      0.33(10)     0.3(2)      Uiso  0.006580 H
   H12d     1.0    0.51(6)      0.33(9)      0.18(18)    Uiso  0.006580 H
   H13d     1.0    0.48(7)      0.34(8)      0.41(18)    Uiso  0.006580 H
   C14d     1.0    0.611(12)    0.524(15)    0.26(3)     Uiso  0.005480 C
   H15d     1.0    0.53(8)      0.59(8)      0.2(2)      Uiso  0.006580 H
   C16d     1.0    0.286(11)    0.573(13)    0.24(3)     Uiso  0.005480 C
   C17d     1.0    0.336(10)    0.670(14)    0.09(3)     Uiso  0.005480 C
   C18d     1.0    0.647(12)    0.449(14)    0.29(3)     Uiso  0.005480 C
   C19d     1.0    0.635(10)    0.584(12)    0.22(3)     Uiso  0.005480 C
   C20d     1.0    0.234(11)    0.621(13)    0.20(3)     Uiso  0.005480 C
   H21d     1.0    0.29(7)      0.52(9)      0.30(19)    Uiso  0.006580 H
   C22d     1.0    0.284(11)    0.718(13)    0.05(3)     Uiso  0.005480 C
   H23d     1.0    0.37(7)      0.69(8)      0.05(19)    Uiso  0.006580 H
   C24d     1.0    0.705(11)    0.438(12)    0.29(3)     Uiso  0.005480 C
   H25d     1.0    0.63(7)      0.41(9)      0.3(2)      Uiso  0.006580 H
   C26d     1.0    0.692(11)    0.574(15)    0.22(3)     Uiso  0.005480 C
   Cl27d    1.0    0.593(3)     0.674(3)     0.182(6)    Uiso  0.008230 Cl
   C28d     1.0    0.233(10)    0.693(13)    0.11(3)     Uiso  0.005480 C
   H29d     1.0    0.20(7)      0.61(9)      0.2(2)      Uiso  0.006580 H
   H30d     1.0    0.28(7)      0.77(9)     -0.0(2)       Uiso  0.006580 H
   C31d     1.0    0.728(10)    0.500(14)    0.26(3)     Uiso  0.005480 C
   H32d     1.0    0.73(7)      0.39(9)      0.3(2)      Uiso  0.006580 H
   H33d     1.0    0.71(9)      0.62(9)      0.2(2)      Uiso  0.006580 H
   C34d     1.0    0.177(12)    0.743(14)    0.08(3)     Uiso  0.005480 C
   H36d     1.0    0.16(8)      0.71(10)     0.02(19)    Uiso  0.006580 H
   H37d     1.0    0.15(7)      0.76(8)      0.21(19)    Uiso  0.006580 H
   H38d     1.0    0.19(7)      0.79(9)     -0.0(2)       Uiso  0.006580 H

#======================================================================
# CRYSTAL DATA
#----------------------------------------------------------------------
data_LT_phase_293K

_chemical_name_common                  'Pigment Orange 34 - LT phase at 293 K'
_cell_length_a                         25.1377(17)
_cell_length_b                         18.4324(16)
_cell_length_c                         7.3554(4)
_cell_angle_alpha                      85.004(3)
_cell_angle_beta                       69.185(6)
_cell_angle_gamma                      70.826(5)
_cell_volume                           3007.254447
_space_group_name_H-M_alt              'P -1'
_space_group_IT_number                 2

loop_
_space_group_symop_operation_xyz
   'x, y, z'
   '-x, -y, -z'

loop_
   _atom_site_label
   _atom_site_occupancy
   _atom_site_fract_x
   _atom_site_fract_y
   _atom_site_fract_z
   _atom_site_adp_type
   _atom_site_U_iso_or_equiv
   _atom_site_type_symbol
   C1a      1.0    0.569(12)    0.003(19)    0.23(4)     Uiso  0.029260 C
   C2a      1.0    0.618(14)   -0.072(19)    0.19(4)     Uiso  0.029260 C
   C3a      1.0    0.596(14)    0.059(19)    0.26(4)     Uiso  0.029260 C
   N4a      1.0    0.511(10)    0.017(15)    0.24(3)     Uiso  0.029260 N
   N5a      1.0    0.666(10)   -0.055(14)    0.20(3)     Uiso  0.029260 N
   O6a      1.0    0.618(8)    -0.139(10)    0.16(2)     Uiso  0.029260 O
   N7a      1.0    0.652(10)    0.025(14)    0.24(3)     Uiso  0.029260 N
   C8a      1.0    0.565(13)    0.14(2)      0.30(4)     Uiso  0.029260 C
   N9a      1.0    0.500(13)   -0.042(13)    0.21(4)     Uiso  0.029260 N
   C10a     1.0    0.725(12)   -0.108(15)    0.17(4)     Uiso  0.029260 C
   H11a     1.0    0.60(9)      0.17(11)     0.3(3)      Uiso  0.035110 H
   H12a     1.0    0.54(10)     0.17(13)     0.2(2)      Uiso  0.035110 H
   H13a     1.0    0.54(9)      0.15(11)     0.4(2)      Uiso  0.035110 H
   C14a     1.0    0.441(13)   -0.029(18)    0.22(4)     Uiso  0.029260 C
   H15a     1.0    0.53(10)    -0.09(12)     0.2(3)      Uiso  0.035110 H
   C16a     1.0    0.748(14)   -0.178(17)    0.06(4)     Uiso  0.029260 C
   C17a     1.0    0.761(14)   -0.085(18)    0.25(4)     Uiso  0.029260 C
   C18a     1.0    0.402(15)    0.046(18)    0.24(5)     Uiso  0.029260 C
   C19a     1.0    0.420(16)   -0.089(15)    0.22(4)     Uiso  0.029260 C
```

```
   C20a      1.0     0.806(15)   -0.225(17)    0.04(4)     Uiso  0.029260 C
   H21a      1.0     0.72(8)     -0.19(13)     0.0(3)      Uiso  0.035110 H
   C22a      1.0     0.818(14)   -0.133(16)    0.22(4)     Uiso  0.029260 C
   H23a      1.0     0.75(9)     -0.04(12)     0.3(2)      Uiso  0.035110 H
   C24a      1.0     0.342(14)    0.058(16)    0.24(5)     Uiso  0.029260 C
   H25a      1.0     0.42(10)     0.09(12)     0.2(3)      Uiso  0.035110 H
   C26a      1.0     0.362(14)   -0.079(18)    0.23(4)     Uiso  0.029260 C
   Cl27a     1.0     0.466(4)    -0.181(5)     0.199(10)   Uiso  0.043880 Cl
   C28a      1.0     0.840(11)   -0.202(17)    0.12(4)     Uiso  0.029260 C
   H29a      1.0     0.82(9)     -0.27(11)     -0.0(3)      Uiso  0.035110 H
   H30a      1.0     0.84(8)     -0.12(11)     0.3(3)      Uiso  0.035110 H
   C31a      1.0     0.322(14)   -0.004(16)    0.24(4)     Uiso  0.029260 C
   H32a      1.0     0.32(9)      0.11(11)     0.3(3)      Uiso  0.035110 H
   H33a      1.0     0.35(9)     -0.12(10)     0.2(3)      Uiso  0.035110 H
   C34a      1.0     0.902(14)   -0.252(16)    0.10(4)     Uiso  0.029260 C
   H36a      1.0     0.91(9)     -0.29(10)     -0.0(2)      Uiso  0.035110 H
   H37a      1.0     0.93(10)    -0.22(12)     0.1(3)      Uiso  0.035110 H
   H38a      1.0     0.90(9)     -0.28(13)     0.2(2)      Uiso  0.035110 H
   C1b       1.0     0.013(14)   -0.005(17)    0.23(4)     Uiso  0.029260 C
   C2b       1.0    -0.035(15)    0.068(16)    0.28(3)     Uiso  0.029260 C
   C3b       1.0    -0.014(13)   -0.058(17)    0.19(4)     Uiso  0.029260 C
   N4b       1.0     0.071(10)   -0.014(13)    0.22(3)     Uiso  0.029260 N
   N5b       1.0    -0.085(11)    0.054(14)    0.27(3)     Uiso  0.029260 N
   O6b       1.0    -0.031(8)     0.129(11)    0.33(2)     Uiso  0.029260 O
   N7b       1.0    -0.070(11)   -0.024(15)    0.22(3)     Uiso  0.029260 N
   C8b       1.0     0.016(14)   -0.142(19)    0.13(4)     Uiso  0.029260 C
   N9b       1.0     0.080(11)    0.046(14)    0.26(4)     Uiso  0.029260 N
   C10b      1.0    -0.145(12)    0.104(18)    0.31(4)     Uiso  0.029260 C
   H11b      1.0    -0.01(10)    -0.16(12)     0.1(3)      Uiso  0.035110 H
   H12b      1.0     0.05(10)    -0.15(11)     0.0(3)      Uiso  0.035110 H
   H13b      1.0     0.03(9)     -0.17(12)     0.2(2)      Uiso  0.035110 H
   C14b      1.0     0.139(14)    0.035(18)    0.26(4)     Uiso  0.029260 C
   H15b      1.0     0.05(10)     0.09(12)     0.3(3)      Uiso  0.035110 H
   C16b      1.0    -0.182(14)    0.079(15)    0.25(3)     Uiso  0.029260 C
   C17b      1.0    -0.169(12)    0.175(18)    0.42(4)     Uiso  0.029260 C
   C18b      1.0     0.186(14)   -0.036(18)    0.19(4)     Uiso  0.029260 C
   C19b      1.0     0.153(12)    0.091(16)    0.32(4)     Uiso  0.029260 C
   C20b      1.0    -0.241(12)    0.123(15)    0.28(4)     Uiso  0.029260 C
   H21b      1.0    -0.17(9)      0.03(11)     0.2(2)      Uiso  0.035110 H
   C22b      1.0    -0.228(14)    0.219(15)    0.46(4)     Uiso  0.029260 C
   H23b      1.0    -0.14(8)      0.19(12)     0.5(3)      Uiso  0.035110 H
   C24b      1.0     0.244(14)   -0.048(18)    0.18(4)     Uiso  0.029260 C
   H25b      1.0     0.18(11)    -0.08(14)     0.1(3)      Uiso  0.035110 H
   C26b      1.0     0.212(14)    0.080(18)    0.32(5)     Uiso  0.029260 C
   Cl27b     1.0     0.100(4)     0.179(5)     0.409(10)   Uiso  0.043880 Cl
   C28b      1.0    -0.263(13)    0.193(17)    0.39(3)     Uiso  0.029260 C
   H29b      1.0    -0.27(10)     0.11(12)     0.2(3)      Uiso  0.035110 H
   H30b      1.0    -0.24(10)     0.27(10)     0.5(2)      Uiso  0.035110 H
   C31b      1.0     0.258(14)    0.009(17)    0.25(5)     Uiso  0.029260 C
   H32b      1.0     0.27(9)     -0.10(12)     0.1(3)      Uiso  0.035110 H
   H33b      1.0     0.22(10)     0.12(11)     0.4(3)      Uiso  0.035110 H
   C34b      1.0    -0.327(13)    0.239(19)    0.43(5)     Uiso  0.029260 C
   H36b      1.0    -0.34(9)      0.24(12)     0.3(2)      Uiso  0.035110 H
   H37b      1.0    -0.35(9)      0.22(12)     0.5(2)      Uiso  0.035110 H
   H38b      1.0    -0.33(9)      0.29(11)     0.5(3)      Uiso  0.035110 H
   C1c       1.0     1.033(17)    0.510(18)    0.25(4)     Uiso  0.029260 C
   C2c       1.0     1.074(12)    0.437(17)    0.28(3)     Uiso  0.029260 C
   C3c       1.0     1.064(14)    0.564(18)    0.24(4)     Uiso  0.029260 C
   N4c       1.0     0.977(11)    0.521(13)    0.24(3)     Uiso  0.029260 N
   N5c       1.0     1.123(10)    0.454(12)    0.28(3)     Uiso  0.029260 N
   O6c       1.0     1.065(8)     0.374(11)    0.30(2)     Uiso  0.029260 O
   N7c       1.0     1.116(11)    0.533(13)    0.26(3)     Uiso  0.029260 N
   C8c       1.0     1.040(13)    0.65(2)      0.21(4)     Uiso  0.029260 C
   N9c       1.0     0.962(12)    0.460(15)    0.26(4)     Uiso  0.029260 N
   C10c      1.0     1.176(14)    0.402(17)    0.30(4)     Uiso  0.029260 C
   H11c      1.0     1.05(10)     0.68(11)     0.3(3)      Uiso  0.035110 H
   H12c      1.0     1.06(10)     0.66(11)     0.1(2)      Uiso  0.035110 H
   H13c      1.0     1.00(8)      0.66(12)     0.2(3)      Uiso  0.035110 H
   C14c      1.0     0.904(14)    0.469(19)    0.26(4)     Uiso  0.029260 C
   H15c      1.0     0.99(10)     0.41(13)     0.3(3)      Uiso  0.035110 H
   C16c      1.0     1.229(15)    0.422(16)    0.23(4)     Uiso  0.029260 C
   C17c      1.0     1.175(14)    0.33(2)      0.40(4)     Uiso  0.029260 C
   C18c      1.0     0.869(16)    0.543(19)    0.22(5)     Uiso  0.029260 C
   C19c      1.0     0.881(13)    0.409(17)    0.29(4)     Uiso  0.029260 C
   C20c      1.0     1.282(13)    0.373(17)    0.26(4)     Uiso  0.029260 C
   H21c      1.0     1.23(10)     0.47(13)     0.2(3)      Uiso  0.035110 H
   C22c      1.0     1.227(14)    0.281(17)    0.43(5)     Uiso  0.029260 C
   H23c      1.0     1.14(9)      0.32(13)     0.4(3)      Uiso  0.035110 H
   C24c      1.0     0.811(16)    0.554(18)    0.22(4)     Uiso  0.029260 C
   H25c      1.0     0.88(9)      0.58(12)     0.2(3)      Uiso  0.035110 H
   C26c      1.0     0.823(13)    0.419(15)    0.29(5)     Uiso  0.029260 C
   Cl27c     1.0     0.923(3)     0.319(5)     0.335(11)   Uiso  0.043880 Cl
   C28c      1.0     1.280(14)    0.303(18)    0.35(4)     Uiso  0.029260 C
   H29c      1.0     1.32(8)      0.39(12)     0.2(3)      Uiso  0.035110 H
   H30c      1.0     1.23(10)     0.23(11)     0.5(2)      Uiso  0.035110 H
   C31c      1.0     0.788(13)    0.493(18)    0.26(4)     Uiso  0.029260 C
   H32c      1.0     0.79(10)     0.60(13)     0.2(3)      Uiso  0.035110 H
   H33c      1.0     0.81(10)     0.38(12)     0.3(3)      Uiso  0.035110 H
   C34c      1.0     1.337(15)    0.25(2)      0.38(5)     Uiso  0.029260 C
   H36c      1.0     1.37(9)      0.27(12)     0.3(2)      Uiso  0.035110 H
   H37c      1.0     1.33(9)      0.25(11)     0.5(2)      Uiso  0.035110 H
   H38c      1.0     1.34(10)     0.20(11)     0.3(3)      Uiso  0.035110 H
   C1d       1.0     0.483(14)    0.496(17)    0.24(4)     Uiso  0.029260 C
   C2d       1.0     0.441(12)    0.568(16)    0.21(4)     Uiso  0.029260 C
   C3d       1.0     0.455(13)    0.438(19)    0.25(4)     Uiso  0.029260 C
   N4d       1.0     0.538(11)    0.486(13)    0.26(3)     Uiso  0.029260 N
   N5d       1.0     0.393(10)    0.548(12)    0.20(3)     Uiso  0.029260 N
   O6d       1.0     0.446(9)     0.633(10)    0.19(2)     Uiso  0.029260 O
   N7d       1.0     0.403(10)    0.467(14)    0.23(3)     Uiso  0.029260 N
   C8d       1.0     0.481(14)    0.355(19)    0.28(4)     Uiso  0.029260 C
   N9d       1.0     0.552(11)    0.546(15)    0.24(4)     Uiso  0.029260 N
   C10d      1.0     0.339(15)    0.598(18)    0.18(4)     Uiso  0.029260 C
   H11d      1.0     0.45(9)      0.33(11)     0.3(3)      Uiso  0.035110 H
   H12d      1.0     0.52(9)      0.33(10)     0.2(2)      Uiso  0.035110 H
   H13d      1.0     0.49(10)     0.35(12)     0.4(3)      Uiso  0.035110 H
   C14d      1.0     0.610(12)    0.532(18)    0.25(4)     Uiso  0.029260 C
   H15d      1.0     0.53(9)      0.59(13)     0.2(3)      Uiso  0.035110 H
   C16d      1.0     0.289(15)    0.571(18)    0.24(4)     Uiso  0.029260 C
   C17d      1.0     0.337(13)    0.671(17)    0.09(4)     Uiso  0.029260 C
   C18d      1.0     0.645(15)    0.459(18)    0.28(4)     Uiso  0.029260 C
   C19d      1.0     0.634(13)    0.591(15)    0.22(4)     Uiso  0.029260 C
   C20d      1.0     0.236(15)    0.618(19)    0.21(4)     Uiso  0.029260 C
   H21d      1.0     0.29(9)      0.52(12)     0.3(3)      Uiso  0.035110 H
   C22d      1.0     0.284(14)    0.718(17)    0.07(4)     Uiso  0.029260 C
   H23d      1.0     0.37(9)      0.69(13)     0.1(3)      Uiso  0.035110 H
   C24d      1.0     0.703(14)    0.446(16)    0.29(5)     Uiso  0.029260 C
   H25d      1.0     0.63(10)     0.42(12)     0.3(3)      Uiso  0.035110 H
   C26d      1.0     0.692(15)    0.580(19)    0.22(4)     Uiso  0.029260 C
   Cl27d     1.0     0.591(3)     0.678(4)     0.179(9)    Uiso  0.043880 Cl
   C28d      1.0     0.234(13)    0.690(17)    0.13(4)     Uiso  0.029260 C
   H29d      1.0     0.20(8)      0.60(16)     0.3(3)      Uiso  0.035110 H
   H30d      1.0     0.28(10)     0.77(13)     0.0(3)      Uiso  0.035110 H
   C31d      1.0     0.726(12)    0.506(19)    0.25(4)     Uiso  0.029260 C
   H32d      1.0     0.73(11)     0.40(13)     0.3(3)      Uiso  0.035110 H
   H33d      1.0     0.71(11)     0.62(12)     0.2(3)      Uiso  0.035110 H
   C34d      1.0     0.177(15)    0.739(19)    0.10(4)     Uiso  0.029260 C
   H36d      1.0     0.16(10)     0.71(13)     0.1(3)      Uiso  0.035110 H
   H37d      1.0     0.15(10)     0.77(11)     0.2(2)      Uiso  0.035110 H
   H38d      1.0     0.19(8)      0.78(14)     0.0(3)      Uiso  0.035110 H

#======================================================================
# CRYSTAL DATA
#----------------------------------------------------------------------
data_HT_phase_493K

_chemical_name_common                  'Pigment Orange 34 - HT phase at 493 K'
_cell_length_a                         11.9124(7)
_cell_length_b                         10.3496(7)
_cell_length_c                         7.3858(4)
_cell_angle_alpha                      108.348(3)
_cell_angle_beta                       88.216(3)
_cell_angle_gamma                      114.226(3)
_cell_volume                           783.105869
_space_group_name_H-M_alt              'P -1'
_space_group_IT_number                 2

loop_
_space_group_symop_operation_xyz
   'x, y, z'
   '-x, -y, -z'

loop_
   _atom_site_label
   _atom_site_occupancy
   _atom_site_fract_x
   _atom_site_fract_y
   _atom_site_fract_z
   _atom_site_adp_type
   _atom_site_U_iso_or_equiv
   _atom_site_type_symbol
   C1        1.0     1.043(6)     0.483(8)     0.770(7)    Uiso  0.086580 C
   C2        1.0     1.143(7)     0.637(6)     0.885(7)    Uiso  0.086580 C
   C3        1.0     1.096(6)     0.378(6)     0.757(7)    Uiso  0.086580 C
   N4        1.0     0.936(4)     0.463(5)     0.704(6)    Uiso  0.086580 N
   N5        1.0     1.237(4)     0.599(6)     0.921(6)    Uiso  0.086580 N
   O6        1.0     1.140(4)     0.766(4)     0.935(5)    Uiso  0.086580 O
   N7        1.0     1.204(4)     0.443(5)     0.841(6)    Uiso  0.086580 N
   C8        1.0     1.039(5)     0.210(7)     0.661(8)    Uiso  0.086580 C
   N9        1.0     0.914(4)     0.584(6)     0.738(6)    Uiso  0.086580 N
   C10       1.0     1.358(6)     0.697(9)     1.027(8)    Uiso  0.086580 C
   H11       1.0     1.04(3)      0.18(3)      0.52(4)     Uiso  0.103900 H
   H12       1.0     1.08(3)      0.17(4)      0.71(5)     Uiso  0.103900 H
   H13       1.0     0.95(3)      0.17(3)      0.69(4)     Uiso  0.103900 H
   C14       1.0     0.799(4)     0.564(8)     0.671(8)    Uiso  0.086580 C
   H15       1.0     0.97(4)      0.68(4)      0.81(5)     Uiso  0.103900 H
   C16       1.0     1.444(8)     0.636(6)     1.023(9)    Uiso  0.086580 C
   C17       1.0     1.386(6)     0.850(10)    1.131(10)   Uiso  0.086580 C
   C18       1.0     0.716(7)     0.418(7)     0.555(10)   Uiso  0.086580 C
   C19       1.0     0.764(5)     0.680(6)     0.718(8)    Uiso  0.086580 C
   C20       1.0     1.560(7)     0.727(9)     1.123(10)   Uiso  0.086580 C
   H21       1.0     1.42(3)      0.53(5)      0.95(5)     Uiso  0.103900 H
   C22       1.0     1.503(7)     0.943(6)     1.232(8)    Uiso  0.086580 C
   H23       1.0     1.33(4)      0.89(6)      1.13(5)     Uiso  0.103900 H
   C24       1.0     0.598(6)     0.393(6)     0.487(7)    Uiso  0.086580 C
   H25       1.0     0.74(3)      0.34(4)      0.52(6)     Uiso  0.103900 H
   C26       1.0     0.649(7)     0.659(8)     0.654(9)    Uiso  0.086580 C
   Cl27      1.0     0.8650(16)   0.8528(16)   0.855(2)    Uiso  0.112560 Cl
   C28       1.0     1.588(6)     0.880(7)     1.227(8)    Uiso  0.086580 C
   H29       1.0     1.62(3)      0.69(6)      1.12(6)     Uiso  0.103900 H
   H30       1.0     1.53(3)      1.05(3)      1.30(4)     Uiso  0.103900 H
   C31       1.0     0.563(3)     0.513(10)    0.536(9)    Uiso  0.086580 C
   H32       1.0     0.54(4)      0.30(4)      0.41(6)     Uiso  0.103900 H
   H33       1.0     0.63(5)      0.74(4)      0.69(5)     Uiso  0.103900 H
   C34       1.0     1.714(6)     0.978(7)     1.332(9)    Uiso  0.086580 C
   H36       1.0     1.77(3)      0.95(4)      1.26(4)     Uiso  0.103900 H
   H37       1.0     1.73(3)      1.08(4)      1.34(5)     Uiso  0.103900 H
   H38       1.0     1.71(3)      0.97(4)      1.46(4)     Uiso  0.103900 H
```
